# Revealing bioremediation potential of novel indigenous bacteria from oil-contaminated sites in the UAE: A combined bioinformatics and experimental validation

**DOI:** 10.1371/journal.pone.0329515

**Published:** 2025-08-12

**Authors:** Sara Awni Alkhatib, Sagar Arya, Deema Islayem, Runyararo Memory Nyadzayo, Sharmarke Mohamed, Ahmed F. Yousef, Hector H. Hernandez, Anna-Maria Pappa

**Affiliations:** 1 Department of Biomedical Engineering and Biotechnology, Khalifa University, Abu Dhabi, United Arab Emirates; 2 Center for Catalysis and Separation (CeCaS), Khalifa University, Abu Dhabi, United Arab Emirates; 3 Department of Chemistry, Green Chemistry & Materials Modelling Laboratory, Khalifa University, Abu Dhabi, United Arab Emirates; 4 Department of Biological Sciences, Khalifa University, Abu Dhabi, United Arab Emirates; 5 Center for Biotechnology (BTC), Khalifa University, Abu Dhabi, United Arab Emirates; 6 Research Center for Membranes and Advanced Water Technology (CMAT), Khalifa University, Abu Dhabi, United Arab Emirates; 7 Tulif Holdings, One Broadway, Cambridge, Massachusetts, United States of America; Mahatma Jyotiba Phule Rohilkhand University, INDIA

## Abstract

Microbial biodegradation of recalcitrant aromatic hydrocarbon pollutants represents an environmentally sustainable strategy for remediating contaminated sites. However, elucidating the metabolic capabilities and genetic determinants of biodegrading strains is crucial for optimizing bioremediation strategies. In this study, we comprehensively characterize the aromatic catabolic potential of two indigenous bacterial isolates, *A. xylosoxidans* C2 (*A. x. C2*) and *A. xylosoxidans* KW38 (*A. x.* KW38), obtained from hydrocarbon-impacted environments in the United Arab Emirates (UAE). Experimental validation through aromatic hydrocarbons supplemented growth studies confirmed the capability of the isolated bacteria to mineralize bisphenol A, 4-hydroxybenzoic acid, 1-naphthalenemethanol, and the high molecular weight polycyclic aromatic hydrocarbon (PAH), pyrene, in the presence of glucose. Their degradation efficiencies were comparable to or greater than those of *Pseudomonas paraeruginosa*, a well-characterized model organism for aromatic compound degradation. Integrated bioinformatic analyses uncovered fundamental aromatic catabolic pathways conserved across *Achromobacter* species, along with strain-specific genes that potentially confer specialized degradative capacities, highlighting the genomic basis of the observed metabolic versatility. Further, protein modeling based on the curated sequences revealed unique features of individual catabolic enzymes and their interaction networks. Notably, a dehydrogenase enzyme involved in aromatic ring cleavage was identified exclusively in these UAE isolates. These findings establish *A. x.* C2 and *A. x.* KW38 as promising bioremediators of diverse aromatic pollutants. Overall, the study exemplifies a powerful and comprehensive methodological framework that bridges bioinformatic analysis and experimental research to further optimize the effectiveness of experimental design. We achieved a substantial reduction in the number of unknown genetic and metabolic determinants of aromatic hydrocarbon degradation in the strains, reducing uncertainty by 99.3%, thereby enhancing the overall process and outcomes for systematic biodiscovery of pollutant-degrading environmental microbes to address ecological challenges.

## Introduction

Aromatic hydrocarbons, including benzene, toluene, ethylbenzene, and xylenes (BTEX), as well as polycyclic aromatic hydrocarbons (PAHs), represent a major class of priority environmental pollutants due to their toxicity, mutagenicity, carcinogenicity, and persistence in contaminated sites [1,2]. The release of these recalcitrant compounds from industrial processes has led to widespread environmental contamination [3]. Owing to their hydrophobic nature and slow degradation rates, BTEXs and PAHs persist in the environment for extended periods, presenting significant risks to human health and ecological systems [4,5]. In pursuit of sustainable remediation strategies, diverse autochthonous microbes and plants are being isolated, studied, and bioengineered for the effective degradation of persistent contaminants like PAHs [1]. Among these, microorganisms possess superior metabolic capabilities for degrading PAHs, converting them into simpler and less toxic metabolites.

Microbes employ diverse enzymatic pathways to catalyze the breakdown of BTEX and PAH compounds, utilizing them as carbon and energy sources [5]. Recent efforts have focused on identifying key microbial degraders by mapping their genomes for aromatic catabolic pathways and optimizing environmental conditions to enhance biodegradation rates [6–16]. Understanding the metabolic machinery and regulatory networks governing pollutant mineralization in microbial strains is crucial for developing and bioengineering effective microbial-driven bioremediation systems. These reported studies provide valuable insights; however, they focus on individual aspects like the identification of certain degradative genes, analyzing the pathways, optimization of environmental conditions, and lack a comprehensive analysis that integrates all these perspectives. This study does not only focus on identifying the genetic and metabolic pathways involved in aromatic hydrocarbon degradation but also integrates a comprehensive methodological framework that bridges the gaps between bioinformatic analysis and experimental validation. This dual approach provides a better understanding of metabolic machinery and regulatory networks in pollutant degradation present in newly identified microbial strains. Our work offers a holistic strategy that elucidates these degradation pathways and sets a robust framework for future studies to develop and bioengineer effective microbial-driven bioremediation systems.

We herein comprehensively characterize the aromatic hydrocarbon degradation potential of two autochthonous bacterial isolates, *Achromobacter* xylosoxidans C2 (*A. x.* C2) and *Achromobacter* xylosoxidans KW38 (*A. x.* KW38), sourced from hydrocarbon-contaminated sites in the United Arab Emirates (UAE). Through phenotypic assays, we confirm their capacity to mineralize a wide range of aromatic pollutants including bisphenol A (BPA), naphthols, hydroxybenzoic acids (HBA), and high molecular weight PAHs as additional carbon sources when supplemented with glucose. Such co-utilization of carbon sources is known to occur in nutritionally complex environments, where glucose serves as the primary carbon source that initiates microbial growth, subsequently inducing genes responsible for the breakdown of aromatic hydrocarbons [17].

To elucidate the genetic basis of their biodegradative versatility, an integrated multi-omics approach combining comparative genomics, metabolic pathway reconstruction, enzyme homology analysis, and protein structural modeling was employed. Mapping their genomic repertoires revealed conserved aromatic catabolic pathways shared across *Achromobacter* species, as well as unique genetic elements potentially contributing to strain-level capability for degrading organic compounds for its use in bioremediation. Furthermore, three-dimensional modeling illuminated the molecular architectures of key degradative enzymes and their interaction networks underlying these complex xenobiotic degradation processes.

This interactomics-based investigation provides a comprehensive systems-level perspective correlates the phenotypic degradation profiles of *A. x.* C2 and *A. x.* KW38 with their genomic and metabolic determinants. The results highlights the biodegradative potential of indigenous Gulf isolates and shed light on the intricate cellular machinery evolved to thrive in polluted ecosystems by harnessing aromatic hydrocarbons. Our findings establish these strains as promising candidates for bioremediation applications while advancing fundamental understanding of microbial xenobiotic catabolism. Furthermore, this study exemplifies a powerful multi-omics workflow to support the biodiscovery of pollutant-degrading environmental microbes to address pressing ecological challenges.

## Methods

### Materials and microorganisms

Two *A.* xylosoxidans strains used in this study were previously isolated from the UAE environment [18] *A. x.* C2 was isolated from soil samples in an old-planted forest in Al Wathbah, Abu Dhabi, UAE and *A. x.* KW38 from oil contaminated sites at the Khorfakkan Port, Sharjah, UAE. Microbial samples were collected from abandoned agricultural lands not under active management or private ownership. These former farmlands are publicly accessible with no access restrictions. No permits were required for sample collection as these sites are not classified as protected areas. The non-native tree species present at the sampling locations are common throughout the UAE and not classified as endangered. No plant material was used in this research. *Pseudomonas paraeruginosa* (ATCC 9027) (*P. paraeruginosa*) by Rudra et al. (2022) [19] and *Escherichia coli* (ATCC 8739) (*E. coli*) were purchased from the American Type Culture Collection (ATCC) (Manassas, VA, USA). All strains were revived from glycerol stock by streak plating on LB agar, followed by incubating at 37 ˚C for 24–48 hrs. Single colonies were picked and grown in LB broth at 37 ˚C for 48 hrs. The culture was subsequently diluted with fresh LB broth to an OD_600_ of 0.2, representing the exponential growth phase, for use in subsequent experiments.

Glucose, LB nutrient broth, LB agar, agar-agar, copper (II) chloride, cobalt (II) chloride hexahydrate, boric acid, manganese (II) chloride, ethylenediaminetetraacetic acid (EDTA), zinc chloride, iron (III) chloride, thiamine-HCl, biotin, calcium chloride dihydrate, magnesium sulfate heptahydrate, di-Sodium hydrogen phosphate dodecahydrate, monopotassium phosphate, sodium chloride, ammonium chloride, sodium hydroxide, analytical grade bisphenol A (BPA), 4-hydroxybenzoic acid (4-HBA), 1-naphthalenemethanol (1-NM), and pyrene (PYR) were purchased from Sigma-Aldrich (Burlington, MA, USA). Acetone and toluene were obtained from Honeywell Inc. (Charlotte, NC, U.S.A).

### Media and carbon source preparation

Minimal salts M9 media and agar plates were prepared according to the manufacturers’ protocol and supplemented with 2 mg/mL glucose [20]. Carbon sources were prepared by solubilizing 2 mg of each of BPA, 4-HBA, and 1-NM in 10 µL 100% acetone. Acetone was allowed to evaporate before adding them to 1 mL of 2 mg/mL of glucose-supplemented media. PYR was similarly prepared by adding 2 mg into 10 µL of 100% toluene, allowing it to evaporate before adding 1 mL of 2 mg/mL of glucose-supplemented media. Each prepared solution was filtered through a 0.22 μm Millipore Express PLUS Stericup® Quick Release-GP Vacuum Filtration System (MilliporeSigma, MA, USA).

### Co-metabolic carbon source optimization

The growth of *A. x.* C2 and *A. x.* KW38 at varying concentrations of glucose and supplementary aromatic carbon sources optimum were monitored at OD_600_ using a NanoDrop™ 2000/2000c spectrophotometer (Thermo Fisher Scientific, Waltham, MA, USA) to determine the optimal glucose-to-aromatic carbon ratio for degradation studies.

The concentration of supplementary glucose was determined by assessing the bacterial growth at four different ratios of glucose to aromatic hydrocarbons. The final concentration of carbon sources (glucose + aromatic hydrocarbons) was maintained constant at 4 mg/mL [21]. The growth was evaluated at varying glucose levels of 0, 1, 2, 3, 4 mg/mL mixed with corresponding aromatic hydrocarbon levels of 4, 3, 2, 1, 0 mg/mL, respectively. The bacterial growth level was determined in each sample after 10 days of incubation using absorbance measurement at OD_600_. The lowest concentration of glucose that initiated the growth was selected as a supplementary carbon source concentration. Subsequently, the maximum concentration of individual aromatic hydrocarbons that the strains can tolerate was tested by growing the strains at increasing concentration of aromatic hydrocarbons (0, 1, 2, 3, 4 mg/mL) in glucose-enriched M9 minimal media [carbon-free M9 minimal media supplemented with 2 mg/mL glucose as the sole additional carbon source].

### Aromatic hydrocarbon degradation assay

An aliquot of 10 µL from each bacterial strain was added into 2 mL of glucose-enriched M9 media containing 2 mg/mL of aromatic hydrocarbons. The samples were incubated in a shaker incubator at 130 rpm and 37 ˚C for 10 days in an orbital incubator/shaker (Model Stuart S1600C, Wagtech, UK). The bacterial growth was determined by measuring the turbidity of the culture at OD_600_ for both bacterial strains on day 10 [22,23].

An aromatic hydrocarbon degradation assay was performed on agar plates to corroborate the results obtained in the liquid culture assay. A total of 1 mL of 2 mg/mL solubilized aromatic hydrocarbon was drop-cast on the surface of agar plate and spread using a sterile spreader, then allowed to evaporate. Once the solvent had evaporated, 100 µL of bacterial culture was spread on the agar plate followed by incubation at 37 ˚C for 10 days.

### Scanning electron microscope (SEM)

The morphology of bacterial cells and colony structures were examined using SEM [24]. Briefly, 10 µL of bacterial culture was diluted to 0.1 OD and grown on a coverslip inside 12-well plate incubated in 37 ˚C incubator. Then the media was gently removed, and the coverslip was washed twice with phosphate buffered saline (PBS) to eliminate any residual contaminants. Then the bacteria were fixed with 2% glutaraldehyde at room temperature for 1 hr. Following fixation, sequential dehydration was performed using 60% ethanol for 10 min, followed by 70%, 80%, 90%, and 100% ethanol. The samples were air-dried, and gold coated for SEM visualization. Similarly, agar slides were prepared by cutting a 1 x 1 cm^2^ piece of agar, followed by fixation and dehydration.

### Mash-based analysis of Acromobacter genomes

At first, Similar Genome Finder from the Bacterial and Viral Bioinformatics Resource Center (BV-BCR), previously known as PATRIC [25] was used to identify genetic distances between the UAE indigenous strains and other members of the *Achromobacter* genus using the Mash/MinHash metric. Genetic distances were calculated using “mash distance”. The analysis resulted in a set of 100 genomes within a p-value threshold = 1, and Mash distance = 1. The results of the Similar Genome Finder were then loaded into a separate genome group, including *A. x.* C2 and *A. x.* KW38 and the 100 selected genomes. A phylogenetic tree analysis was conducted on the list of genomes, obtained from the Similar Genome Finder, using the Codon Tree method [26] and were performed using 500 genes for analysis, and zero allowed deletions and duplications (results of 1000 genes are similar).

### Selected study group for comparative analysis

Six closely related *Achromobacter* species: *A. arsenitoxydan*s SY8, *A. piechaudii* ATCC 43553, *A.* sp. DMS1, *A. xylosoxidans* A8, *A. xylosoxidans* subsp. *xylosoxidans* NCTC 10807, and *A. denitrificans* NBRC 15125 genomes along with *A. x.* C2 and *A. x.* KW38 were selected for comparative genomic analysis. Complete genomes of selected *Achromobacter* species were retrieved into the BV-BRC database.

### Identifying enzymes involved in degradation of aromatic hydrocarbons

Annotation of putative aromatic carbon degradation pathways was performed for all 6 selected species bacteria using BV-BRC. Next, pathway information about adjacent enzymes was extracted from and pathway neighbors were defined as enzymes that are connected to each other through a common substrate. Using BLAST and the Kyoto Encyclopedia of Genes and Genomes (KEGG) database, we mapped coding sequences (CDS) into similar enzymes (<99% identity) in reference genomes and extracted EC numbers from reference genomes. Metabolic pathways absent in both *A. x.* C2 and *A. x.* KW38 were excluded from the analysis.

The putative enzyme functions analysis was performed manually by comparing selected enzyme domains to known protein structures in reference genomes using Pymol (pymol.org) [27] and BV-BRC. Multiple sequence alignment (MSA) was performed for each of the degradation enzymes separately to compare and manually curate the data before threading their structures.

We performed a global protein alignment using BV-BRC MSA tool. In some instances, there were multiple copies of the enzyme identified in the genomes. Protein sequences below 100 amino acids, sequences longer than the average length of occurrences, similar sequences (containing sequence members of different families), and alignments with inserts greater than 10 amino acids were excluded from the analysis. Additional MSA was performed using T-Coffee (tcoffee.crg.eu) [28] and MUSCLE alignment tools (ebi.ac.uk) [29]. Moreover, genome search *via* BLAST (blast.ncbi.nlm.nih.gov) [30] was performed using UniProt (uniprot.org) [31] and NCBI on the following species: *Achromobacter, Acidovorax, Verminephrobacter, Comamanas, Variovorax, Ramlibacter, Hydrogenophaga, Rhizobacter, Pelomonas, Rubrivivax, Azohydromonas, Sphaerotilus, Thiomonas, Bordetella, Castellaniella, Bordetella, Pusillimonas, Candidatus, Burkholderia, Pandoraea, Capriavidus, Ralstonia, Cupriavidus, Massilia, Janthinobacterium, Massilia, Herbaspirillum, Collimonas, Thauera, Azoarcus, Dechlorosoma, Pseudoglubenkiania, Xanthomonas, Stenotrophomonas, Lysobacter, Arenimonas, Pseudomonas,* and *Sphingomonas*-like bacterium. The results were aligned to the *A. x.* C2 and *A. x.* KW38 genomes to assist in refining gene structures.

### Protein structure prediction by threading methods

All selected enzymes were passed through the Search Tool for the Retrieval of Interacting Genes (STRING) (string-db.org) [32] to obtain their protein-protein interactions (PPIs) network connection. STRING integrates known and predicted interactions from multiple sources. However, such computational predictions may include false positives or lack context-specific information, highlighting the inherent challenges in accurately interpreting PPI networks. Therefore, screening of the PPI network was performed to select the highest connected module with the core proteins having greater than 98% identity to proteins in *A.xylosoxidans* NBRC15126.

Secondary structure prediction was performed by threading the amino acid sequences to the library of known folds using Jpred4 [33], Phyre2 [34], and PSIPRED (bio.tools/psipred) [35,36]. The program PyMOL was used for visualization and comparison of protein structure alignments [37]. All enzymes were uploaded to the Search Tool for the Retrieval of Interacting Genes (STRING) [32] to construct the PPI network with no clustering option was chosen. Screening of the constructed modules was performed to select the highest connected module. Only proteins that had > 98% identity to proteins in *A.xylosoxidans* NBRC 15126 were used in the analysis.

### Data analysis

All data obtained in the study were subjected to a t-test, p < 0.05 was considered statistically significant. The absorbance readings in the optimization of carbon source and the biodegradation assay represent the bacterial growth as the mean ± SD of three replicas. This article does not contain any studies with human participants or animals performed by any of the authors.

## Results and discussion

**Fig 1:**
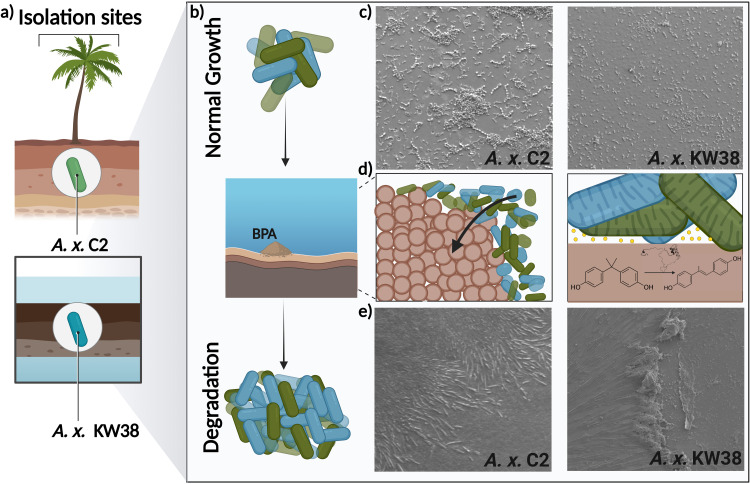
Analyzing the aromatic hydrocarbon degradation potential of two UAE-indigenous strains. **a.** Schematic diagram representing the isolation sites of *A*. *x.* C2 and *A.* *x.* KW38 from UAE. b. Schematic diagram representing the increase in growth levels of *A.* *x.* C2 and *A.* *x.* KW38 when exposed to aromatic hydrocarbons (e.g., BPA). **c.** SEM images of *A.* *x.* C2 and *A.* *x.* KW38 grown in glucose for 24 hrs at 37 °C without BPA. **d**. Schematic representing the initial step in the enzymatic degradation of BPA to 4,4’-dihydroxy-a-methystilbene in the two strains, full degradation pathway in S12 Fig **in** S1 File. **e.** SEM images of *A.* *x.* C2 and *A. x.* KW38 grown in a BPA containing media for 10 days at 37 ˚C, indicating their growth and organization within this environment. Figure created using BioRender.com [38].

Our study follows a systematic approach to analyze the aromatic hydrocarbon bioremediation potential of the two strains that were previously sourced by our team from environmentally challenging sites in the UAE, Fig 1 [18]. Upon isolation, we first validated the degradation capabilities of these strains, hypothesizing that these strains possessed the capacity to degrade aromatic structures, Fig 1b,1d. The logic behind testing the degradation potential of these two bacterial strains was twofold; *A. x.* C2, isolated from soils, is likely to be an oil-degrading strain that produces amphiphilic molecules to solubilize oil [39–41], and *A. x.* KW38 isolated from an oil spill sample is expected to be an oil-degrading bacterial strain [42–44]. We confirmed their degradation capability through two distinct degradation assays: an aqueous-based assay that mimics the degradation process occurring in freshwater environment and solid-based assay that emulates the degradation in soil. Subsequent to these experimental validations, we conducted a comprehensive bioinformatic analysis of the two bacterial strains which identified the principal metabolic degradation pathways encoded in their genomes along with the corresponding enzymes associated with each pathway. A comparative analysis was conducted to compare these strains with their closest phylogenetic relatives. Placed in the context of these genomes, the genetic framework for determining the metabolic variations and similarities that do exist within *Achromobacter* related to aromatic hydrocarbon degradation metabolism is analyzed. Additionally, we present the three-dimensional structures of the identified enzymes, offering valuable insights into their molecular structures and the degree of interaction among them is elucidated in a PPI network.

### Optimizing growth conditions of A. x. C2 and A. x. KW38 in presence of aromatic hydrocarbons

**Fig 2:**
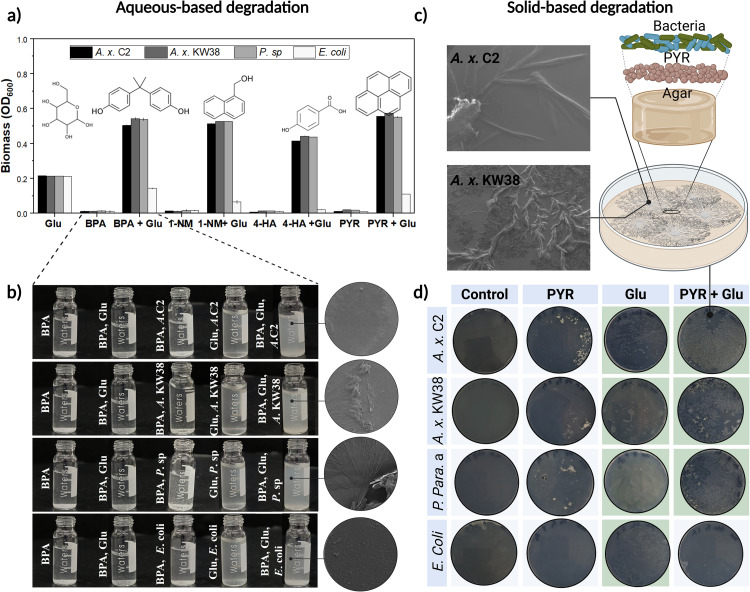
Evaluating biodegradation of aromatic hydrocarbons by *A.* ***x.* C2, *A. x.* KW38, *P. paraeruginosa*, and *E. coli* under optimized growth conditions**. Incubation conditions: 37 ˚C, pH 7.0, shaking at 130 rpm both in aqueous and solid-based degradation assays. **a.** Absorbance measurements quantifying bacterial growth after 10 days in M9 minimal media with 2 mg/mL glucose and 2 mg/mL of the indicated aromatic hydrocarbon. Chemical structures are shown. Error bars represent standard deviation (n = 9). **b.** Visual turbidity assay comparing growth of the four strains in BPA, glucose, and BPA + glucose conditions supported with SEM images. **c.** Schematic illustrating the solid agar assay setup with glucose and aromatic hydrocarbons coated onto the surface before adding bacterial cultures, supported with SEM images showing growth in form of fractal structures surrounding insoluble PYR particles on the agar surface. **d.** glucose-enriched agar plates, streaked with the four strains in the presence of different aromatic hydrocarbons and incubated for 10 days at 37 ˚C. Triplicates validate degradation potential, particularly for PYR. colors describing the scoring system for growth (in green), no growth (in blue) on the solid agar assay plates Figure created using BioRender.com [45].

Prior to the biodegradation assays, growth optimization experiments were conducted to determine the optimal growth conditions for *A. x.* C2 and *A. x.* KW38 in the presence of aromatic hydrocarbons. We observed that the bacterial strains used for aromatic hydrocarbon degradation did not exhibit significant growth when cultured directly on the aromatic compounds, Fig 2a, 2b). Supplementing the culture with the minimum amount of co-carbon source, like glucose, stimulated initial microbial growth and further induce the expression of degradation genes required to utilize the aromatic compounds [46–48]. In various bacteria, the consumption of aromatic compounds like benzene, toluene, and phenanthrene is supported by the initial availability of glucose, which subsequently activate key enzymes including catechol 1,2-dioxygenase, benzene/toluene/chlorobenzene dioxygenase, and phenanthrene monooxygenases, S3 Table in S1 File. The shift from glucose to aromatic compounds generally requires the activation of certain degradation pathways, facilitating the effective microbial adaptation and breakdown of aromatic compounds in environments with various carbon sources [49,50]. Therefore, the strains were cultivated in carbon-free M9 minimal media supplemented with various concentrations of glucose as a supplementary carbon source to determine the optimal growth conditions for subsequent degradation of BPA, 4-HBA, 1-NM, and PYR, S1 Fig in S1 File. The minimum threshold required to support microbial activity was 2 mg glucose-C/g soil. At lower glucose concentrations, the supplemental glucose was insufficient to initiate the bacterial growth. Our findings were consistent with the results reported in the study of Stephanie Reischke et al., 2015 [51], where they found that only concentrations of 2 mg glucose-C/g soil and higher met the energy demand for maintaining active biomass and resulted in an exponential increase in bacterial respiration.

The effects of glucose concentration on growth were determined by testing glucose: aromatic hydrocarbon ratios of (4:0, 3:1, 2:2, 1:3, and 0:4) with a final concentration of both carbon sources at 2 mg/mL. For all four aromatics, the 1:1 ratio (2 mg/mL glucose) supported optimal growth after 10 days, as indicated by OD_600_, see S1a–S1d Fig in S1 File. Lower glucose levels did not permit robust growth, likely due to aromatic inhibition, while higher levels elevated turbidity independently of aromatic catabolism. *P*. *paraeruginosa* (positive control) and *E*. *coli* (negative control) followed similar trends, though *E*. *coli* grew less, potentially from aromatic toxicity [52].

Keeping glucose concentration at 2 mg/mL, the individual effect of aromatic concentration was tested in the range of (0–4 mg/mL). For BPA, 4-HBA, 1-NM, and PYR, growth decreased progressively with increasing aromatic levels above 2 mg/mL, with complete inhibition at 4 mg/mL, S1e–S1h Fig in S1 File. Therefore, 2 mg/mL was selected as the optimized aromatic concentration, supporting moderate growth (average OD_600_ ≈ 0.2) suitable for biodegradation studies. At 2 mg/mL of aromatics, the growth level of *A. x.* C2 and *A. x.* KW38 closely matched the level of *P*. *paraeruginosa* while *E*. *coli* grew slightly less, likely lacking efficient aromatic catabolism. In summary, supplementing the media with equal amounts of glucose and aromatic hydrocarbons (2 mg/mL each) resulted in a robust growth of *A. x.* C2 and *A. x.* KW38 comparable to *P. paraeruginosa*.

### Exploring aromatic hydrocarbon degradation in the UAE-isolated bacterial strains, A. x. C2 and A. x. KW38

The bacterial strains *A. x.* C2 and *A. x.* KW38 were previously isolated from surface rhizosphere of desert plants and oil-contaminated site, respectively, as shown in Fig 1a. Given their isolation from ecologically degraded and contaminated environments, we looked at their potential to degrade aromatic hydrocarbons using both solid and aqueous-based culture assays. Previous work identified *A. x.* C2 and *A. x.* KW38 as gram-negative aerobic strains [18]. The SEM images Fig 1c revealed that *A. x.* C2 and *A. x.* KW38 are rod shaped, non-flagellated bacteria with a wrinkled, rough, and glossy surface. The cell dimensions ranged from 1.1–1.9 µm in length and 0.5–0.8 µm in width.

In the solid-based assay, agar plates, supplemented with 2 mg/mL glucose, were surface coated with solubilized aromatic hydrocarbons. The solvent was allowed to evaporate before introducing the fresh bacterial cultures. In the liquid assay, soluble aromatic hydrocarbons served as the sole carbon source in M9 minimal media, with 2 mg/mL of glucose provided as a supplementary carbon source. The growth level of both *A. x.* C2 and *A. x.* KW38 was promoted in these two assays compared with the growth level in glucose alone, indicating the ability of the strains to metabolize the respective aromatic compounds and thrive more in their presence, Fig 1b **and** 1e). *P. paraeruginosa*, a well-characterized aromatic hydrocarbon degrader, was used as a positive control [53–58], while *E. coli*, which lacks natural aromatic catabolic capabilities, served as the negative control [59].

Both strains exhibited growth levels comparable to *P. paraeruginosa* when cultured in M9 media abundant with aromatic hydrocarbons, as well as on agar coated with aromatic hydrocarbons. The aqueous-based assay, Fig 2a **and** 2b), demonstrated high growth level in the presence of aromatics compared to glucose alone for degrading strains *A. x.* C2, *A. x.* KW38, and *P. paraeruginosa* (supplementary figures S2–S4 Figs **in** S1 File for other aromatics). Likewise, the growth on agar showed results as evidenced by the similar colony size and number, Fig 2d, (supplementary figures S5–S8 Figs **in** S1 File for other aromatics). This suggests the potential ability of these strains to degrade the supplied aromatic substances in a performance resembling the degradative potential of *Pseudomonas*. In contrast, *E*. *coli* showed negligible growth in both solid and liquid media with aromatic hydrocarbons, as expected. However, it showed low growth level in BPA and PYR, these results support the findings reported by Bruce S. Hass et al., where BP was found to promote the growth of E. *coli*, and PYR has little to no effect on its growth [59].

These results show the capacity of the indigenous UAE strains *A. x.* C2 and *A. x.* KW38 to degrade a range of aromatic hydrocarbons through putative dedicated catabolic pathways. Their growth in the presence of these compounds highlights their metabolic capabilities and their potential use in bioremediation of organic aromatic pollutants.

Growth on aqueous and solid media revealed distinct degradation profiles for the four bacterial strains when challenged with different aromatic hydrocarbons in the presence of glucose as a co-metabolic substrate. For BPA, the strain *A. x.* KW38 and the model degrader, *P. paraeruginosa* displayed the highest growth capabilities based on culture turbidity, agar plate colonies, and biomass accumulation, S5 Fig in S1 File. This suggests that these strains possess efficient catabolic pathways and tolerance mechanisms to mineralize BPA as a primary carbon and energy source when supplemented with glucose. In contrast, *E*. *coli* exhibited moderate growth inhibition by BPA, only achieving partial degradation likely through general metabolic pathways instead of dedicated ones. Some engineered *E*. *coli* strains can degrade BPA via introduced bisdA and bisdB genes, but the wild-type strain used here lacked such specialized functions [60].

A divergent pattern emerged with 1-NM, where *A. x.* C2, *A. x.* KW38, and *P*. *paraeruginosa* demonstrated comparable and robust growth, while *E*. *coli* proliferation was completely abolished, S2 and S6 Figs in S1 File. This stark inhibition aligns with previous studies reporting *E*. *coli*’s extreme sensitivity to even trace 1-NM levels (0.05 mg/mL). The UAE isolates thus appear to possess distinct enzymatic machinery conferring tolerance and metabolic processing of this naphthol compound that *E*. *coli* naturally lacks.

For 4-HBA, S3 and S7 Figs in S1 File, all three degrader strains (*A. x.* C2, *A. x.* KW38, *P*. *paraeruginosa*) displayed significantly reduced growth compared to other aromatics. This decrease in substrate degradation over the time course of the experiment may relate to the structural features of 4-HBA, which can induce multidrug efflux pumps and unidentified resistance mechanisms in *E*. *coli* that completely inhibited its proliferation. Other aromatic acids like benzoic acid are known to act as potent antimicrobials against *E*. *coli* and other gram-negative pathogens [52].

The high-ring aromatic hydrocarbon PYR proved challenging for most strains except *A. x.* C2 and *A. x.* KW38, which effectively tolerated and grew on 2 mg/mL in both liquid and solid assays (S4 and S8 Figs in S1 File). In contrast, *E*. *coli* was unable to proliferate at this elevated PYR concentration. While some reports describe engineered PYR-degrading *E*. *coli*, the wild-type strain used here lacked intrinsic tolerance/catabolic capabilities for this heavily condensed polycyclic aromatic structure. *P*. *paraeruginosa* also showed reduced growth on PYR compared to the other aromatics tested. The robust degradation by the UAE isolates highlights their potential for remediating PYR and other high molecular weight PAH pollutants.

Interestingly, all three aromatic degraders (*A. x.* C2, *A. x.* KW38, *P*. *paraeruginosa*) formed distinctive fractal biofilm patterns surrounding insoluble aromatic clusters on the solid agar plates, Fig 2d. This unique morphology reflects the adaptive biological phenomena which is visual evidence of the ability of the aromatic degraders to utilize catabolic pathways and diffusion gradients to access, funnel, and mineralize solid-phase aromatic hydrocarbon pollutants as carbon/energy sources once supplemental glucose is depleted. Fractal biofilms are usually observed under conditions of diffusion limitation or nutrient gradients because they help maximize surface area and increase efficiency of expansion and resource capture. According to T. Vicsek et al. [61], when bacterial growth is dominated by slowly spreading nutrients they grow in diffusion-limited aggregation forming fractal structures. Furthermore, the presence of such complex morphologies provides evidence that the degraders are undergoing advanced catabolic mechanisms to utilize limited carbon sources. In other words, as glucose gets depleted, the branching growth spreads more to increase the metabolic reach and access to the insoluble clusters of aromatic hydrocarbons. Such phenomena has also been explored in other studies in which bacterial biofilm formed fractal patterns as a response to nutrient limitation [62–64].

Various mechanisms may contribute to bacterial survival when utilizing PAHs as a carbon source. Certain bacteria operate within microbial communities, producing exopolysaccharides that house enzymes capable of degrading PAHs. Specifically, in the case of *A. xylosoxidans*, research indicates that it releases biosurfactants, which enhance the bacteria’s ability to adhere to and break down PAHs effectively [65]. Additionally, other studies suggest that bacteria secrete specialized polysaccharides that facilitate efficient diffusive-based transport of PAHs from crystalline forms to bacterial cells [66]. Moreover, among the bacteria examined, biofilm formation on PAH sources emerged as a key strategy to address mass transfer challenges associated with poorly soluble PAHs. The synthesis of surfactants can lower both surface tension and interfacial tension, a process known as surfactant-enhanced remediation (SER) [67]. Based on this, we can propose that the combination of biosurfactant production and biofilm formation may be the primary mechanisms driving bacterial attachment to and utilization of PAHs in our study. Nevertheless, further multiomics investigations are needed to elucidate the precise molecular responses underlying these processes.

Although the UAE indigenous isolates *A. x.* C2 and *A. x.* KW38 demonstrated versatile degradation capacities across all aromatics tested, there were notable differences in their efficiencies relative to *P. paraeruginosa* and *E*. *coli* which correlated with compound structures and strains’ intrinsic metabolic networks. These results highlight their promise as bioremediators, but also reveal substrate-specific variations that warrant further investigation of underlying enzymatic and regulatory mechanisms governing their aromatic catabolic metabolism.

### Interactomics investigation: Mapping molecular interactions through genome dynamics and comparative analysis

To validate the results of the degradation assays, subsequent bioinformatic analysis was utilized to determine the available metabolic pathways and specific enzymes that can be potentially active during the degradation of aromatic hydrocarbons. Initially, these strains went through high throughput sequencing and characterization processes [18]. Genome sequencing and annotation revealed that *A. x.* C2 and *A. x.* KW38 as closely related *Achromobacter* strains with 6.55 Mbp and 6.58 Mbp genomes, ~ 68% GC content, and 10 rRNA operons each [18]. To investigate their aromatic hydrocarbon degradation capabilities, a multi-omics bioinformatic analysis pipeline was employed, S9 Fig in S1 File. We developed a comprehensive methodological framework that strategically locates the pathways and enzymes involved in the degradation of aromatic hydrocarbons. The framework is based on integrating several key steps to systematically search for specific metabolic pathways and proteins, analyzing their sequences and structures to optimally improve future experimental work and design.

Comparative analysis via BLAST revealed significant sequence and functional similarity between the two strains. The current investigation focuses on a systematic methodology designed to assess the biodegradation capabilities of these bacterial strains involving aromatic hydrocarbon structures and identifying the metabolic pathways and specific enzymes associated with aromatic hydrocarbon degradation within their sequences.

**Fig 3:**
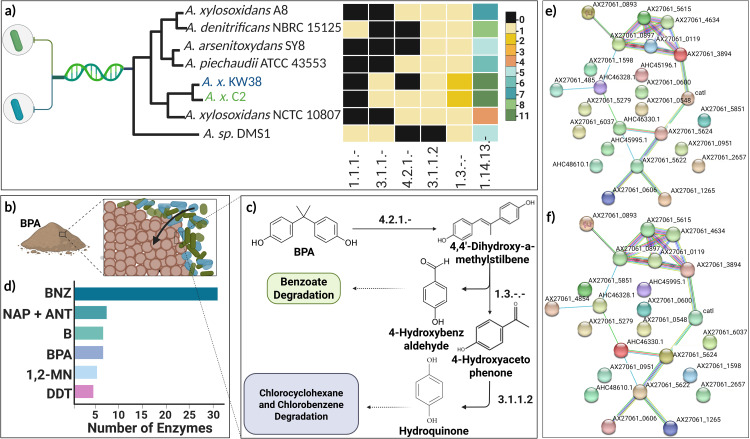
Comparative bioinformatic analysis, including phylogenetic, enzymatic, and PPI network analysis of BPA-degrading UAE-indigenous bacterial strains. **a.** Phylogenetic tree analysis of UAE-indigenous strains (in blue and green) and other bacterial strains globally available from other regions (full phylogenetic tree is available in S10 Fig **in** S1 File) and the corresponding enzyme heatmap representing the number of occurrences of each enzyme involved in BPA breakdown pathway in all selected strains. **b.** Schematic representing the degradation of BPA using *Achromobacter* strain. **c.** The corresponding BPA degradation pathway active during the breakdown of structure highlighting the EC numbers of enzymes involved in each step. **d.** The number of enzymes present in each aromatic hydrocarbon degradation pathways in all the genomes analyzed in the phylogenetic tree (Fig 3a). **e.** PPI network complex and modular analysis of 25 curated enzymes that degrade aromatic hydrocarbons in *A.* ***x.*** KW38, PPI: number of nodes = 25, number of edges = 21, average node degree = 1.68, average local clustering coefficient = 0.353. **f.** PPI network complex for A. x. C2, PPI; number of nodes = 24, number of edges = 21, average node degree = 1.75, average local clustering coefficient = 0.368. *Here in Fig 3e **and** 3f), network nodes represent proteins, splice isoforms or post-translational modifications are collapsed, i.e., each node represents all the proteins produced by a single, protein-coding gene locus. Figure created using BioRender.com [38].

Initially, a BLAST-based homology search against 500 public bacterial genomes identified six *Achromobacter* strains as closest relatives to *A. x.* C2 and *A. x.* KW38 based on whole genome distance estimation using Mash/MinHash approaches. Phylogenomic analysis positioned the UAE isolates in a distinct clade alongside these six reference strains (*A.* xylosoxidans A8, *A.* denitrificans NBRC 15125, *A.* arsenitoxydans SY8, *A.* piechaudii ATCC 43553, *A.* xylosoxidans NCTC 10807, *A.* *sp.* DMS 1), which were included for intraspecies comparative genomics, Fig 3a, details available in S1 Table in S1 File.

Global pathway mapping revealed 6 out of 140 total pathways (details are available in S2 Table in S1 File) directly involved in aromatic hydrocarbon/PAH degradation: naphthalene/anthracene (NAP/ANT) (S11 Fig in S1 File), PAH (S12 Fig in S1 File), dioxin (S13 Fig in S1 File), BP (Fig 3c and S14 Fig in S1 File), benzoate hydroxylation (BNZ) (S15 Fig in S1 File), and dichlorodiphenyltrichloroethane (DDT) pathway (S16 Fig in S1 File). An additional 16 lipid metabolism pathways contributing to biosurfactant synthesis were found to indirectly support aromatic catabolism. Within these six core degradation routes, 37 unique enzyme entries were identified (details of each are available in S3 Table in S1 File), 25 of which (66%) were conserved across the studied *Achromobacter* genomes. Interestingly, one dehydrogenase (EC 1.14.13.1) was exclusively present in *A. x.* C2 and *A. x.* KW38. In contrast, 11 enzymes were unique to other reference strains. The benzoate hydroxylation pathway contained 80% of the total aromatic degradation enzymes mapped (details available in S18 and S22 Figs in S1 File). Despite overall conservation, manual curation of enzyme sequences unveiled strain-specific variations. Pairwise alignment analyses identified *A.* xylosoxidans NCTC 10807 as most closely related to the UAE isolates based on enzyme repertoires, while *A.* sp. DMS1 was the most divergent. Most enzymes yielded high-quality alignments (scores 995–1000) after multi-step sequence curation (details of each are available in S4 Table). Subsequently, the protein structures were threaded into 3D structures. The structures of the 25 enzymes are available in the supplementary document S23–S48 Figs in S1 File).

Structural modeling enabled construction of PPI networks for 25 curated aromatic degradation enzymes in *A. x.* KW38 and 24 in *A. x.* C2, Fig 3e and 3f). These interconnected networks were significantly enriched for biological processes related to aromatic catabolism like the beta-ketoadipate pathway. Certain enzymes served as network hubs potentially interacting with multiple partners, S18–S22 Figs in S1 File. The PPI network topology, Fig 3e and 3f), together with the genomic evidence of conserved aromatic catabolic pathways shared across *Achromobacter* species, substantiates the phenotypic observations of *A. x.* C2 and *A. x.* KW38 efficiently mineralizing diverse aromatic pollutants. Unique genetic elements like the exclusive dehydrogenase further point to specialized features underpinning these strains’ biodegradative versatility. In summary, this integrated multi-omics approach leveraging comparative genomics, pathway reconstruction, enzyme homology, and interactome mapping has provided a comprehensive systems-level perspective, correlating the indigenous isolates’ remarkable aromatic hydrocarbon degradation capabilities with their genomic and metabolic machinery. The insights gained highlighted their promising potential for future biotechnological applications in bioremediation and waste treatment.

## Conclusion

This comprehensive study has provided compelling evidence that the indigenous bacterial strains *A. x.* C2 and *A. x.* KW38, isolated from hydrocarbon-contaminated environments in the UAE, possess remarkable capabilities for degrading a wide range of aromatic pollutants. Through an integrative approach combining phenotypic assays and multi-omics analyses, we have characterized the metabolic and genomic bases underpinning their biodegradative versatility. Aqueous and solid-phase growth assays demonstrated the strains’ proficiencies in utilizing BPA, 1-NM, 4-HBA, and PYR as sole carbon and energy sources when supplemented with minimal glucose concentrations. Their degradation efficiencies matched or exceeded those of the model aromatic degrader *P*. *paraeruginosa*, while outperforming the non-degrader *E*. *coli*. Distinct colonization patterns, with fractal biofilm structures forming around insoluble aromatic clusters, provided visual evidence of their abilities to access and funnel these recalcitrant pollutants.

Comparative genomic analyses revealed extensive repertoires of aromatic catabolic pathways and enzymes conserved across *Achromobacter* species yet exhibited strain-specific variations potentially contributing to metabolic specializations. The mapped key degradation routes included those targeting NAP, ANT, benzoates, BPA, B, and MN. The benzoate degradation pathway emerged as a central aromatic catabolic hub. Protein structural modeling further elucidated potential interactions within intricate enzyme networks driving these biodegradative processes. Notably, a unique dehydrogenase enzyme involved in hydroxylated aromatic ring cleavage was identified exclusively in *A. x.* C2 and *A. x.* KW38, underscoring their distinctive genetic elements. This specialized enzyme, together with specific variations across their cohort of aromatic oxygenases, dehydrogenases, and hydrolases, likely underpins the observed substrate preferences and efficiencies relative to other strains.

The results highlight how these UAE isolates have evolved robust metabolic networks enabling them to thrive in polluted ecosystems by harnessing aromatic hydrocarbons as nutrient sources. Their efficacy at degrading high molecular weight PAHs like PYR are particularly promising for bioremediation applications targeting recalcitrant polyaromatic contaminants. Nevertheless, additional LC/MS-based investigations are warranted to quantify the degradation and pinpoint the byproducts released after bioremediation.

In conclusion, this study addresses longstanding challenges in biochemical research which is the elucidation of intricate protein networks that have potential for certain required chemistries. We provided a comprehensive methodological framework that strategically overcomes these knowledge gaps and optimizing experimental work and design by bridging between bioinformatic analysis and experimental research. The current research achieved a substantial reduction in the number of unknown genetic and metabolic determinants of aromatic hydrocarbon degradation process in *A. x.* C2 and *A. x.* KW38 to a refined 4% uncertainty level. The number of unknown enzymes reduced by 99.3% (from 3476 to 25 for *A. x.* KW38, and from 3482 to 24 for *A. x.* C2) which thus reduces the streamlined experimental work and enhances the investigation process. The core of our methodology is the thorough sequencing of proteins, especially the unexplored ones, and 3D structural modeling. Employing this methodology enabled us to disclose the promising biodegradative protein candidates and prioritize them based on relevance.

Overall, our methodology combining both phenotypic and molecular level studies confirmed the isolated bacteria as promising bioremediators while highlighting key elements of their catabolic machinery that could be further optimized through genetic engineering with implications in polluted site restoration, industrial waste treatment, and biological sensing of aromatic pollutants. Moreover, this work establishes an integrated systems biotechnology workflow for mapping xenobiotic degradation pathways in environmental isolates, facilitating future biodiscovery efforts.

## Supporting information

S1 File Appendix S-1. Aqueous-based degradation assay S1 Fig. Optimizing carbon source levels (aromatic hydrocarbons and glucose as a supplementary carbon source to initiate bacterial growth). S2 Fig. The turbidity of aqueous-based assays employed to visually evaluate the bacterial growth level in 4-HBA for four bacterial strains under different study conditions. S3 Fig. The turbidity of aqueous-based assays employed to visually evaluate the bacterial growth level in PYR for four bacterial strains under different study conditions. Appendix 2. Solid-based degradation assay. S4 Fig. Solid-based assay for BPA degradation using different bacterial strains on carbon-free agar plates under different study conditions. **Appendix S-2. Solid-Based Degradation Assay:** S5 Fig. Solid-based assay for 1-NM degradation using different bacterial strains on carbon-free agar plates under different study conditions. S6 Fig. Solid-based assay for 4-HBA degradation using different bacterial strains on carbon-free agar plates under different study conditions. S7 Fig. Solid-based assay for PYR degradation using different bacterial strains on carbon-free agar plates under different study conditions. S8 Fig. Solid-based assay for PYR degradation using different bacterial strains on carbon-free agar plates under different study conditions. **Appendix S-3. Phylogenetic Tree Analysis:** S9 Fig. Schematic flowchart of the comprehensive methodology used in this study (bioinformatics pipeline developed). S10 Fig. Full phylogenetic tree for A. x. C2 and A. x. KW38. **Appendix S-4. Aromatic Hydrocarbon Degradation Pathways of Strains A. X. A. C2 and A. X. KW38:** S11 Fig. NAP degradation pathway map00626 for A. x. C2 and A. x. KW38. S12 Fig. PAH degradation pathway map00624 for A. x. C2 and A. x. KW38. S13 Fig. Dioxin degradation pathway map00621 for A. x. C2 and A. x. KW38. S14 Fig. BP degradation pathway map00363 for A. x. C2 and A. x. KW38. S15 Fig. BNZ degradation pathway map00362 for A. x. C2 and A. x. KW38. S16 Fig. DDT degradation pathway map00351 for A. x. C2 and A. x. KW38. **Appendix S-5. Selected Genomes and Associated Metadata:** S1 Table. The list of genomes in the selected study group for comparative analysis including UAE indigenous strains and the six other closest relatives from the phylogenetic tree analysis. **Appendix S-6. Aromatic Hydrocarbon Degradation Pathways and Associated Enzymes:** S2 Table. List of pathways available in the selected genome group, the selected pathways are highlighted in grey. S3 Table. The list of enzymes involved in the degradation of aromatic structures available in the selected study group. S4 Table. Details of the selected enzymes for this study and the sequence curation details for every enzyme. **Appendix S-7. Enzyme Heatmaps:** S17 Fig. Distribution of enzymes related to BNZ degradation via hydroxylation pathway (map00362) in 8 genomes. S18 Fig. Distribution of enzymes related to 1,1,1-Trichloro-2,2-bis(4-chlorophenyl)ethane (DDT) degradation (map00351) in 8 genomes. S19 Fig. Distribution of enzymes related to BPA degradation (map00363) in 8 genomes. S20 Fig. Distribution of enzymes related to B degradation (map00621) in 8 genomes. Figure S21. Distribution of enzymes related to MN degradation (map00624) in 8 genomes. S22 Fig. Distribution of enzymes related to NAP and ANT degradation (map00626) in 8 genomes. **Appendix S-8. Threaded enzyme structures:** S23 Fig. Visualization of the final predicted structure of enzyme (EC.1.1.1.1) in a. A. x. C2 and b. A. x. KW38. S24 Fig. Visualization of the final predicted structure of enzyme (EC.1.14.12.3) in a. A. x. C2 and b. A. x. KW38. S25 Fig. Visualization of the final predicted structure of enzyme (EC.1.14.13.-) in a. A. x. C2 and b. A. x. KW38. S26 Fig. Visualization of the final predicted structure of enzyme (EC. 1.14.13.1) in a. A. x. C2 and b. A. x. KW38. S27 Fig. Visualization of the final predicted structure of enzyme (EC. 1.14.13.24) in a. A. x. C2 and b. A. x. KW38. S28 Fig. Visualization of the final predicted structure of enzyme (EC.1.2.1.32) in a. A. x. C2 and b. A. x. KW38. S29 Fig. Visualization of the final predicted structure of enzyme (EC.1.3.-.-) in a. A. x. C2 and b. A. x. KW38. S30 Fig. Visualization of the final predicted structure of enzyme (EC.2.1.1.-) in A. x. KW38. S31 Fig. Visualization of the final predicted structure of enzyme (EC.2.3.1.-) in a. A. x. C2 and b. A. x. KW38. S32 Fig. Visualization of the final predicted structure of enzyme (EC.2.3.1.16) in a. A. x. C2 and b. A. x. KW38. S33 Fig. Visualization of the final predicted structure of enzyme (EC.2.3.1.174) in a. A. x. C2 and b. A. x. KW38. S34 Fig. Visualization of the final predicted structure of enzyme (EC.2.3.1.9) in a. A. x. C2 and b. A. x. KW38. S35 Fig. Visualization of the final predicted structure of enzyme (EC.2.8.3.6) in a. A. x. C2 and b. A. x. KW38. S36 Fig. Visualization of the final predicted structure of enzyme (EC.3.1.1.-) in a. A. x. C2 and b. A. x. KW38. S37 Fig. Visualization of the final predicted structure of enzyme (EC.3.1.1.2) in a. A. x.C2 and b. A. x. KW38. S38 Fig. Visualization of the final predicted structure of enzyme (EC.3.1.1.24) in a. A. x. C2 and b. A. x. KW38. S39 Fig. Visualization of the final predicted structure of enzyme (EC.3.1.1.57) in a. A. x. C2 and b. A. x. KW38. S40 Fig. Visualization of the final predicted structure of enzyme (EC.3.1.2.23) in a. A. x. C2 and b. A. x. KW38. S41 Fig. Visualization of the final predicted structure of enzyme (EC.3.7.1.9) in a. A. x. C2 and b. A. x. KW38. S42 Fig. Visualization of the final predicted structure of enzyme (EC.4.1.1.44) in a. A. x. C2 and b. A. x. KW38. S43 Fig. Visualization of the final predicted structure of enzyme (EC.4.2.1.-) in A. x. C2. S44 Fig. Visualization of the final predicted structure of enzyme (EC.4.2.1.17) in a. A. x. C2 and b. A. x. KW38. S45 Fig. Visualization of the final predicted structure of enzyme (EC. 5.3.3.4) in a. A. x. C2 and b. A. x. KW38. S46 Fig. Visualization of the final predicted structure of enzyme (EC. 5.5.1.1) in a. A. x. C2 and b. A. x. KW38. S47 Fig. Visualization of the final predicted structure of enzyme (EC.5.5.1.2) in a. A. x. C2 and b. A. x. KW38. S48 Fig. Visualization of the final predicted structure of enzyme (EC.6.2.1.25) in a. A. x. C2 and b. A. x. KW38.
(DOCX)
