# Supplementary material for: Revealing bioremediation potential of novel indigenous bacteria from oil-contaminated sites in the UAE: A combined bioinformatics and experimental validation
Source: PLoS One. 2025 Aug 12;20(8):e0329515. doi: 10.1371/journal.pone.0329515 (PMC12342319; doi:10.1371/journal.pone.0329515)
Supplement: S1 File — Optimizing carbon source levels (aromatic hydrocarbons and glucose as a supplementary carbon source to initiate bacterial growth). S2 Fig. The turbidity of aqueous-based assays employed to visually evaluate the bacterial growth level in 4-HBA for four bacterial strains under different study conditions. S3 Fig. The turbidity of aqueous-based assays employed to visually evaluate the bacterial growth level in PYR for four bacterial strains under different study conditions. Appendix 2. Solid-based degradation assay. S4 Fig. Solid-based assay for BPA degradation using different bacterial strains on carbon-free agar plates under different study conditions. Appendix S-2. Solid-Based Degradation Assay: S5 Fig. Solid-based assay for 1-NM degradation using different bacterial strains on carbon-free agar plates under different study conditions. S6 Fig. Solid-based assay for 4-HBA degradation using different bacterial strains on carbon-free agar plates under different study conditions. S7 Fig. Solid-based assay for PYR degradation using different bacterial strains on carbon-free agar plates under different study conditions. S8 Fig. Solid-based assay for PYR degradation using different bacterial strains on carbon-free agar plates under different study conditions. Appendix S-3. Phylogenetic Tree Analysis: S9 Fig. Schematic flowchart of the comprehensive methodology used in this study (bioinformatics pipeline developed). S10 Fig. Full phylogenetic tree for A. x. C2 and A. x. KW38. Appendix S-4. Aromatic Hydrocarbon Degradation Pathways of Strains A. X. A. C2 and A. X. KW38: S11 Fig. NAP degradation pathway map00626 for A. x. C2 and A. x. KW38. S12 Fig. PAH degradation pathway map00624 for A. x. C2 and A. x. KW38. S13 Fig. Dioxin degradation pathway map00621 for A. x. C2 and A. x. KW38. S14 Fig. BP degradation pathway map00363 for A. x. C2 and A. x. KW38. S15 Fig. BNZ degradation pathway map00362 for A. x. C2 and A. x. KW38. S16 Fig. DDT degradation pathway map00351 for A. x. C2 an [file pone.0329515.s001.docx]

**Supplementary Information**

**Revealing bioremediation potential of novel indigenous bacteria from oil-contaminated sites in the UAE: a combined bioinformatics and experimental validation**

Sara Awni Alkhatib^1,2^, Sagar Arya^1#a^, Deema Islayem^1^, Runyararo Memory Nyadzayo^1^, Sharmarke Mohamed^3^, Ahmed F. Yousef^4,5,6^, Hector H. Hernandez^1,7*^, Anna-Maria Pappa^1,2,5*^

Emails: {100058924}@ku.ac.ae {arya}@vutbr.cz {l00042995}@ku.ac.ae {memohove}@gmail.com {sharmarke.mohamed}@ku.ac.ae {ahmed.yousef}@ku.ac.ae {hector.hernandez}@tulif.org, {anna.pappa}@ku.ac.ae

^1^ Department of Biomedical Engineering and Biotechnology, Khalifa University, Abu Dhabi, P.O. Box 127788, United Arab Emirates

^2^ Center for Catalysis and Separation (CeCaS), Khalifa University, Abu Dhabi, P.O. Box 127788, United Arab Emirates

^3^ Department of Chemistry, Green Chemistry & Materials Modelling Laboratory, Khalifa University, Abu Dhabi, P.O. Box 127788, United Arab Emirates

^4^ Department of Biological Sciences, Khalifa University, Abu Dhabi, P.O. Box 127788, United Arab Emirates

^5^ Center for Biotechnology (BTC), Khalifa University, Abu Dhabi, P.O. Box 127788, United Arab Emirates

^6^ Research Center for Membranes and Advanced Water Technology (CMAT), Khalifa University, Abu Dhabi, P.O. Box 127788, United Arab Emirates

^7^ Tulif Holdings, One Broadway, Cambridge, Massachusetts, 1 Broadway Street, Cambridge MA 02139, United States of America

^#a^Current Address: Czech Advanced Technology and Research Institute (CATRIN), Palacký University Olomouc, Šlechtitelů 27, 77900, Olomouc, Czech Republic

* Corresponding author

E-mail: hector.hernandez@tulif.org, anna.pappa@ku.ac.ae

**S-1. Aqueous-based degradation assay**


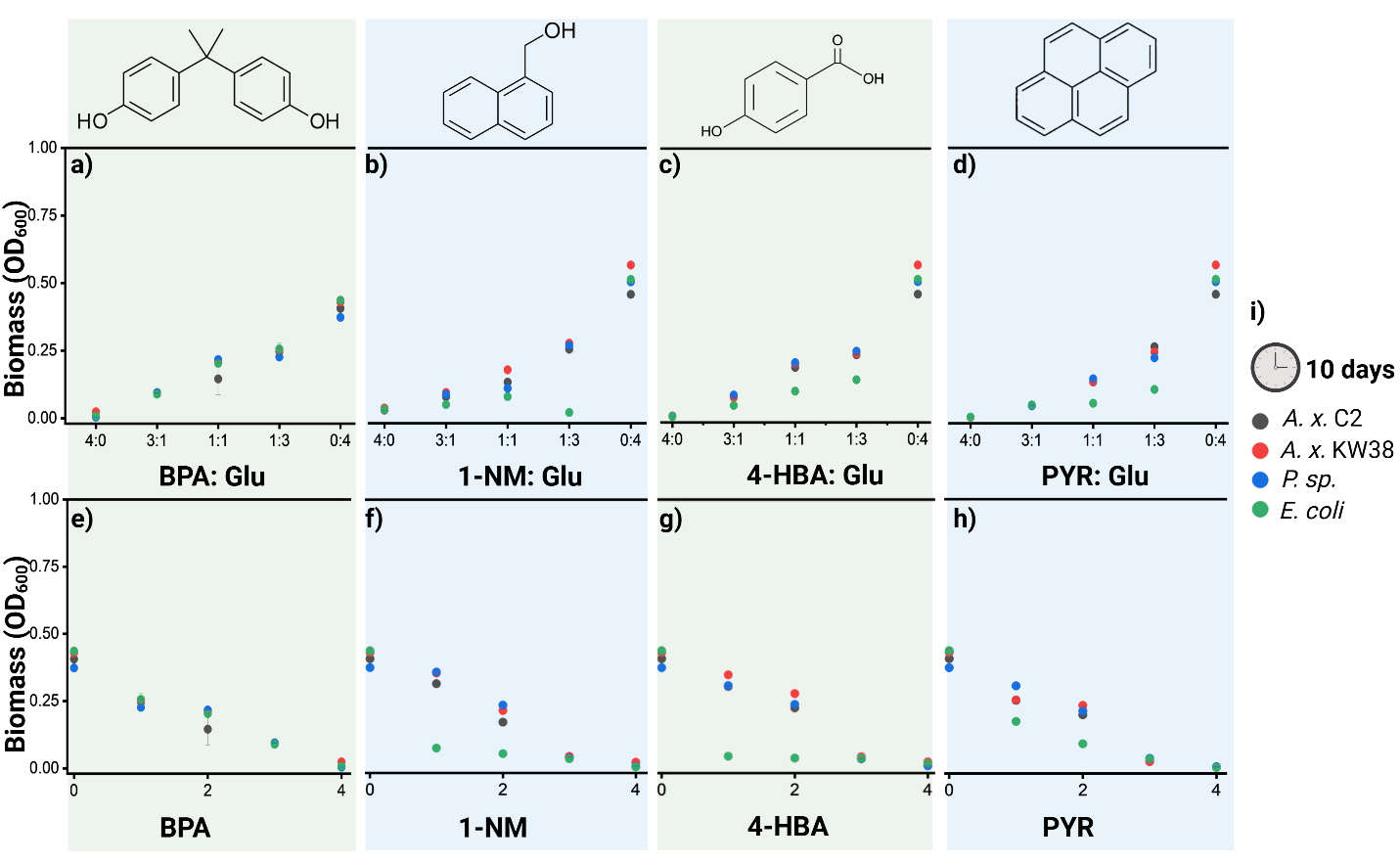


**Figure S1**. **Optimizing carbon source levels (aromatic hydrocarbons and glucose as a supplementary carbon source to initiate bacterial growth (a-d)** shows the absorbance of the bacterial growth at 600 nm after 10 days of incubation under different aromatic hydrocarbon to glucose ratios. **(e-h)** shows the absorbance of the different bacterial strains at 600 nm under different concentrations of aromatic hydrocarbons at fixed glucose level of 2 mg/ml, pH of 7.0 after 10 days of incubation at 37 ˚C, and 130 rpm. Error bars represent deviation (n=3). (**i).** Graphs legend and incubation time. Figure created using biorender.com [41].


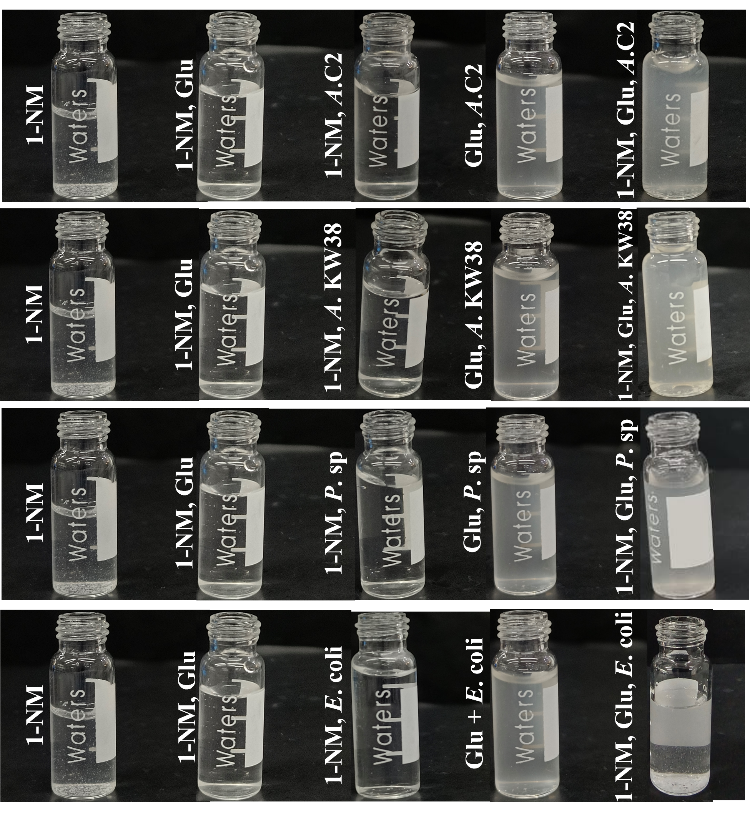


**Figure S2.** The turbidity of aqueous-based assays employed to visually evaluate the bacterial growth level in 1-NM for four bacterial strains under different study conditions.


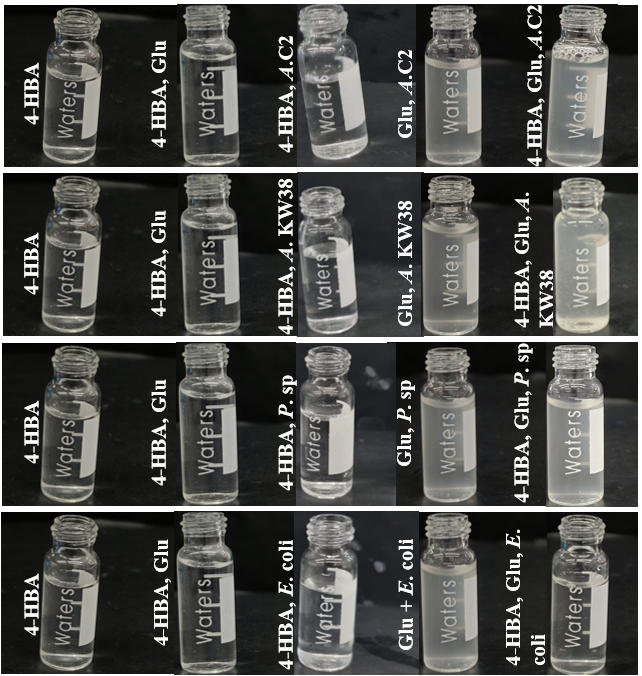


**Figure S3.** The turbidity of aqueous-based assays employed to visually evaluate the bacterial growth level in 4-HBA for four bacterial strains under different study conditions.


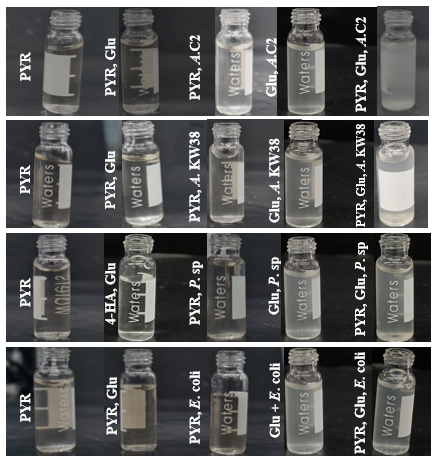


**Figure S4.** The turbidity of aqueous-based assays employed to visually evaluate the bacterial growth level in PYR for four bacterial strains under different study conditions.

**S-2. Solid-based degradation assay**

**
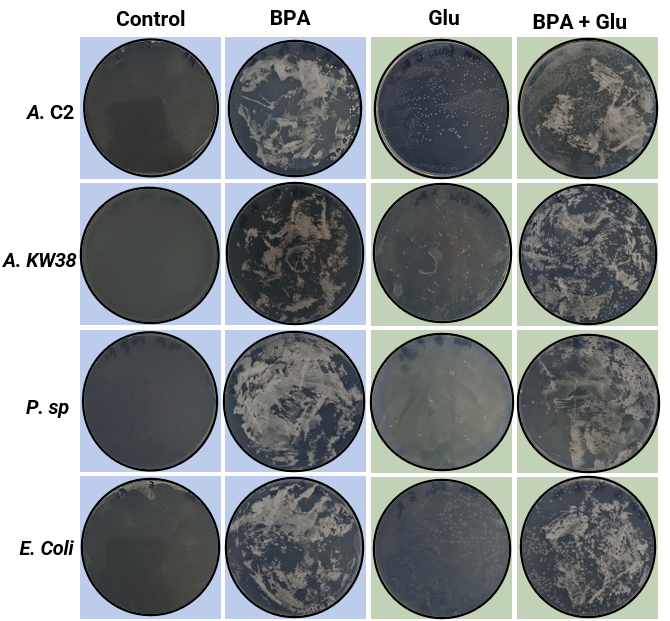
**

**Figure S5.** **Solid-based assay for BPA degradation using different bacterial strains on carbon-free agar plates under different study conditions.** Control contains only agar, plates with glucose are supplemented with 2mg/ml glucose. In green, are plates with growth, in blue, are plates with no growth after 10 days.


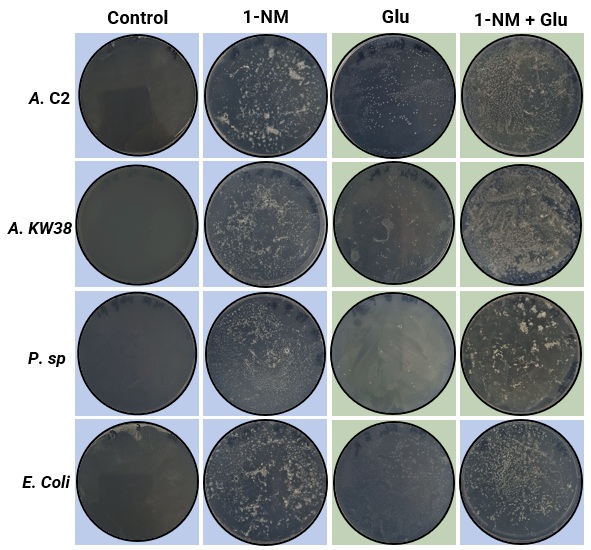


**Figure S6. Solid-based assay for 1-NM degradation using different bacterial strains on carbon-free agar plates under different study conditions.** Control contains only agar, plates with glucose are supplemented with 2mg/ml glucose. In green, are plates with growth, in blue, are plates with no growth after 10 days.


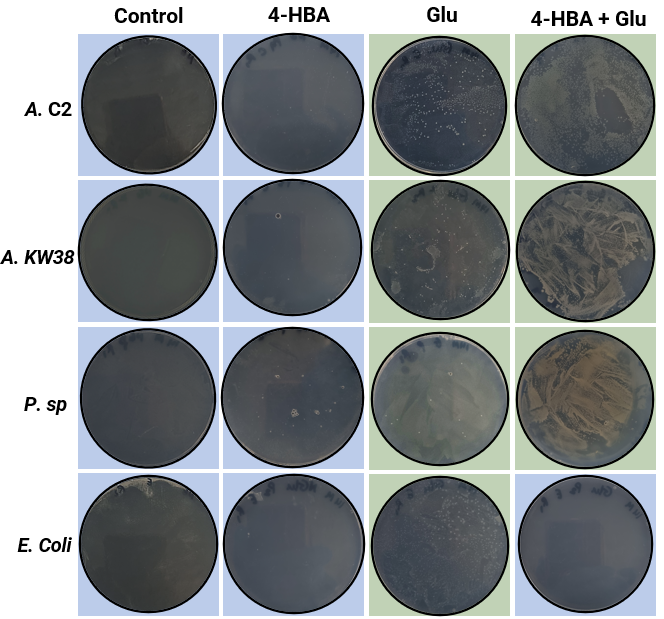


**Figure S7.** **Solid-based assay for 4-HBA degradation using different bacterial strains on carbon-free agar plates under different study conditions.**  Control contains only agar, plates with glucose are supplemented with 2mg/ml glucose. In green, are plates with growth, in blue, are plates with no growth after 10 days.


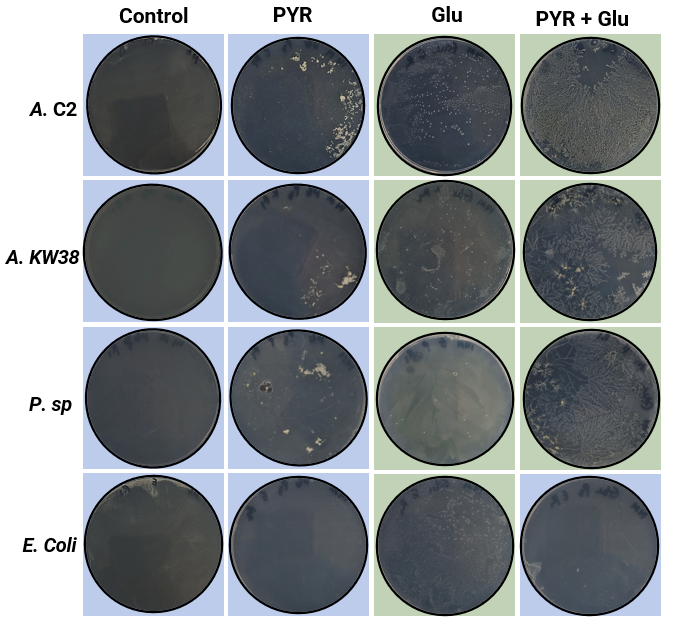


**Figure S8.** **Solid-based assay for PYR degradation using different bacterial strains on carbon-free agar plates under different study conditions.** Control contains only agar, plates with glucose are supplemented with 2mg/ml glucose. In green, are plates with growth, in blue, are plates with no growth after 10 days.

**S-3. P****hylogenetic tree analysis**


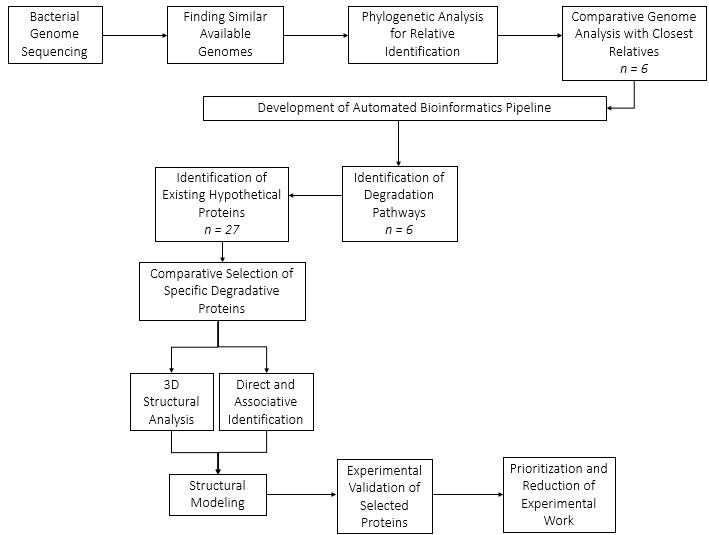


**Figure S9.** Schematic flowchart representing the comprehensive methodology used in this study (bioinformatics pipeline developed)


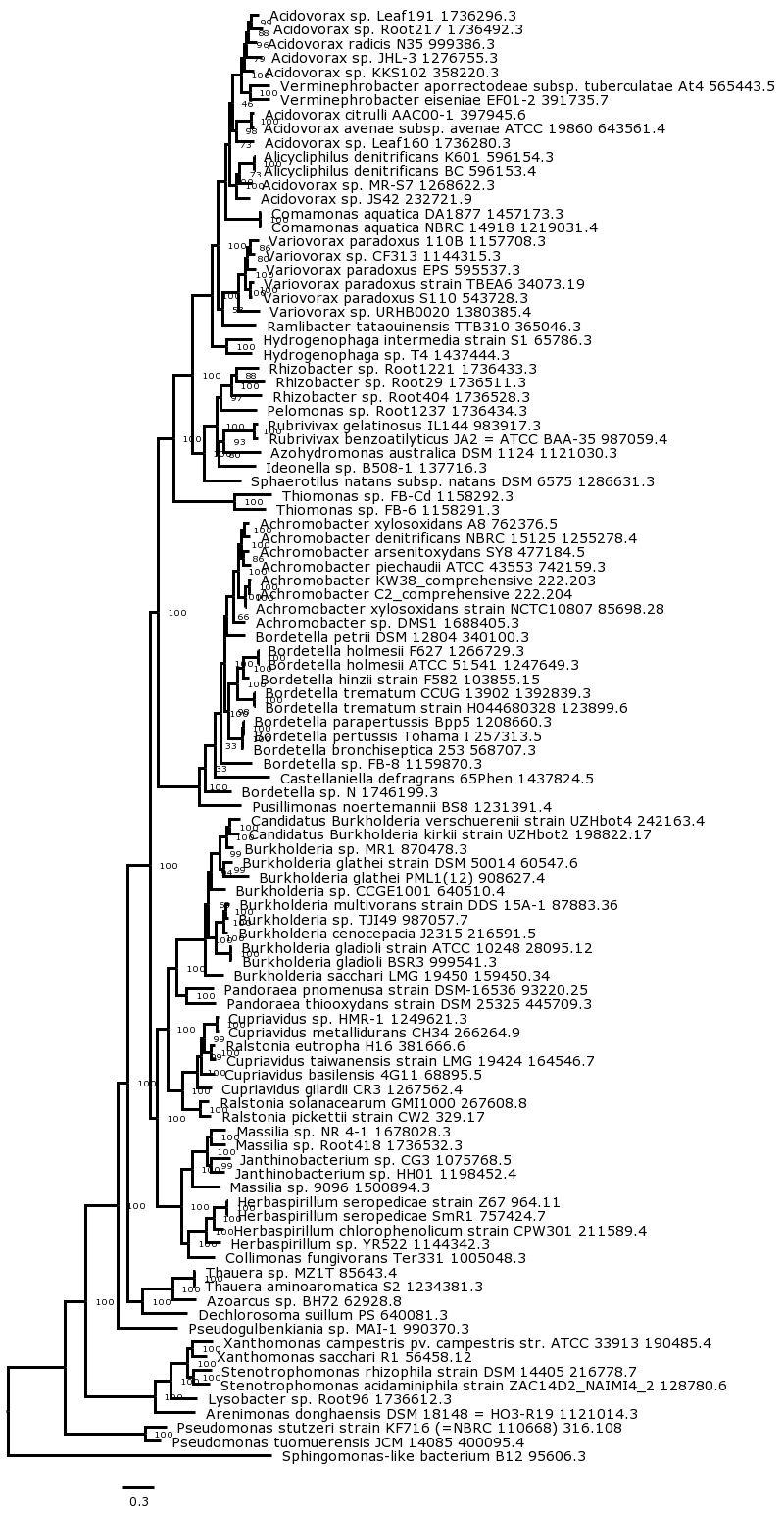


**Figure S10.** Full phylogenetic tree for A. x. C2 and A. x. KW38.

**S-4. Aromatic hydrocarbon degradation pathways of strains *A. X.* *A.* C2 and *A. X.* KW38**

Metabolic pathways are reproduced from BV-BCR ; the presence of enzymes in the *A. x.* C2 and *A. x.* KW38 genomes is shown by colored boxes according to the legend in Figure A1. , indicating the completeness of the pathway. The legend for all metabolic pathway figures (Fig. S11–S16) is as follows: white indicates not annotated, light green indicates annotated, and pale green indicates presence in some genomes


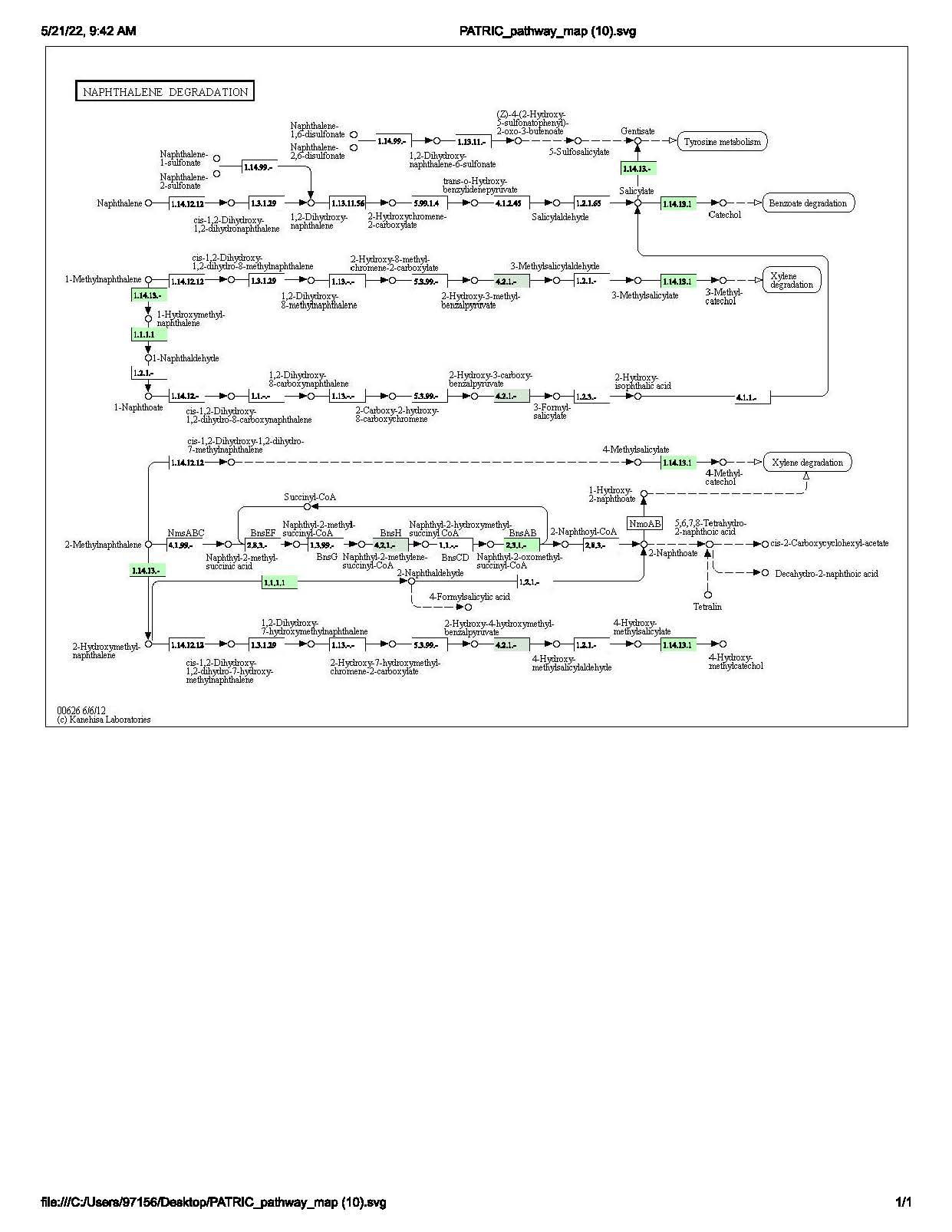


**Figure S11.** NAP degradation pathway map00626 for A. x. C2 and A. x. KW38.


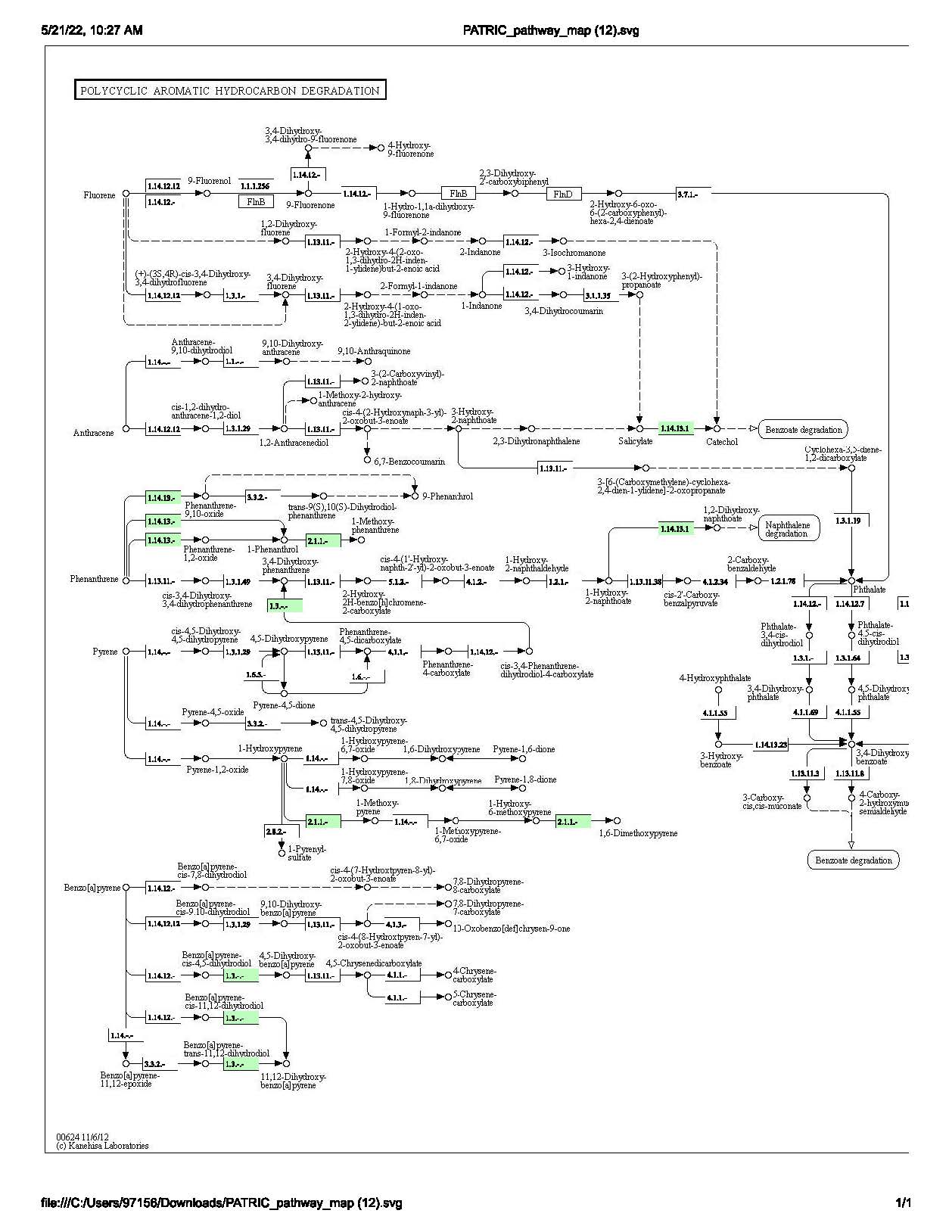


**Figure S12.** PAH degradation pathway map00624 for A. x. C2 and A. x. KW38.


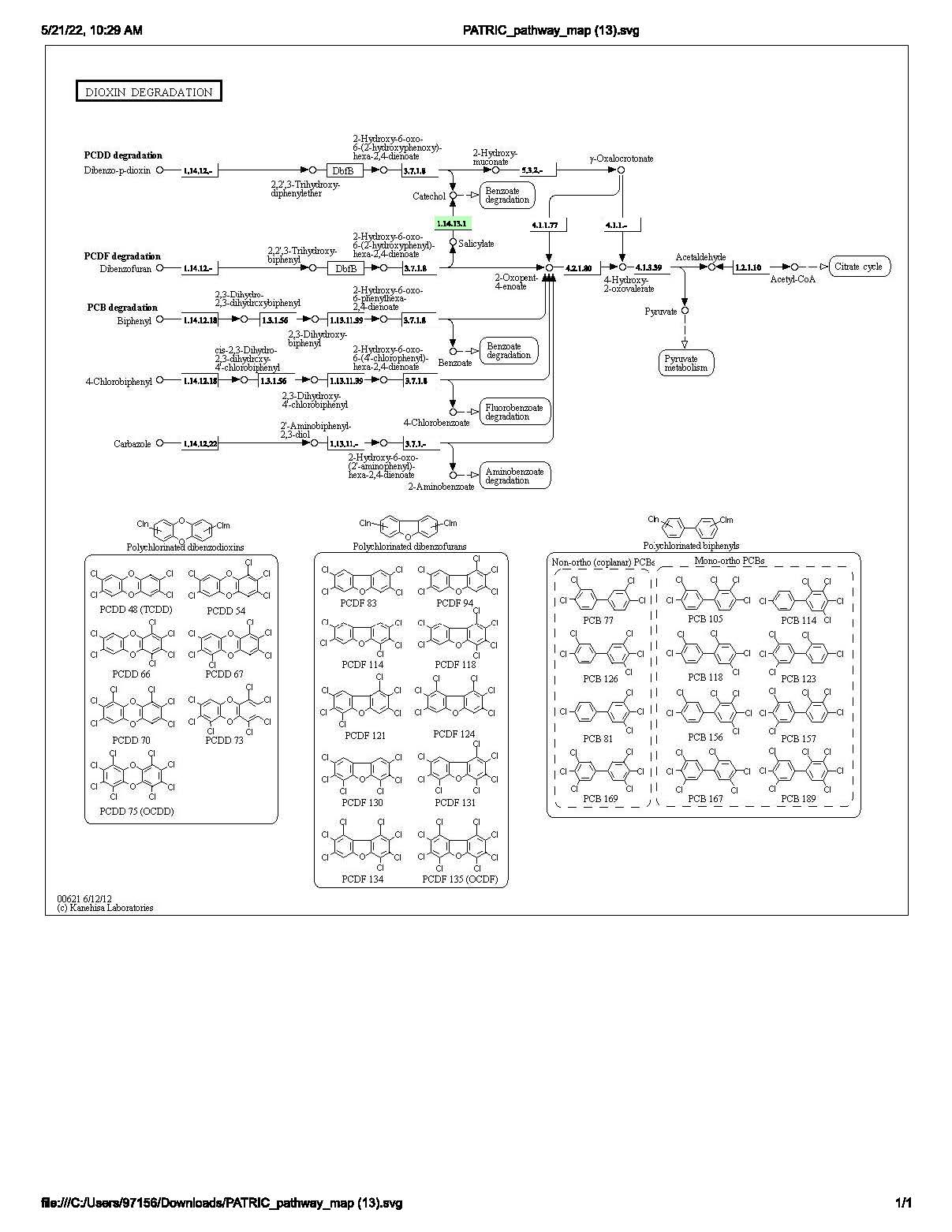


**Figure S13.** Dioxin degradation pathway map00621 for A. x. C2 and A. x. KW38.


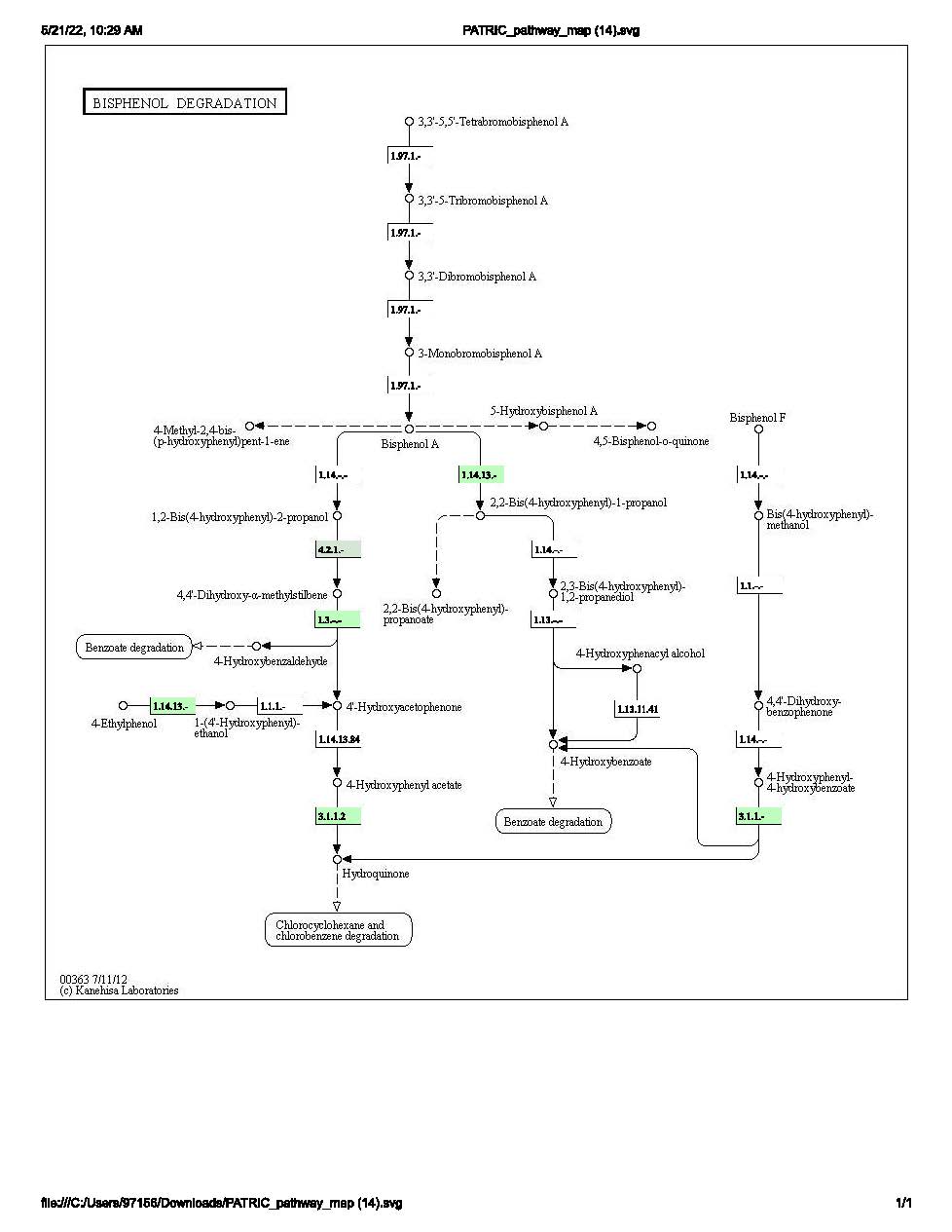


**Figure S14.** BP degradation pathway map00363 for A. x. C2 and A. x. KW38.


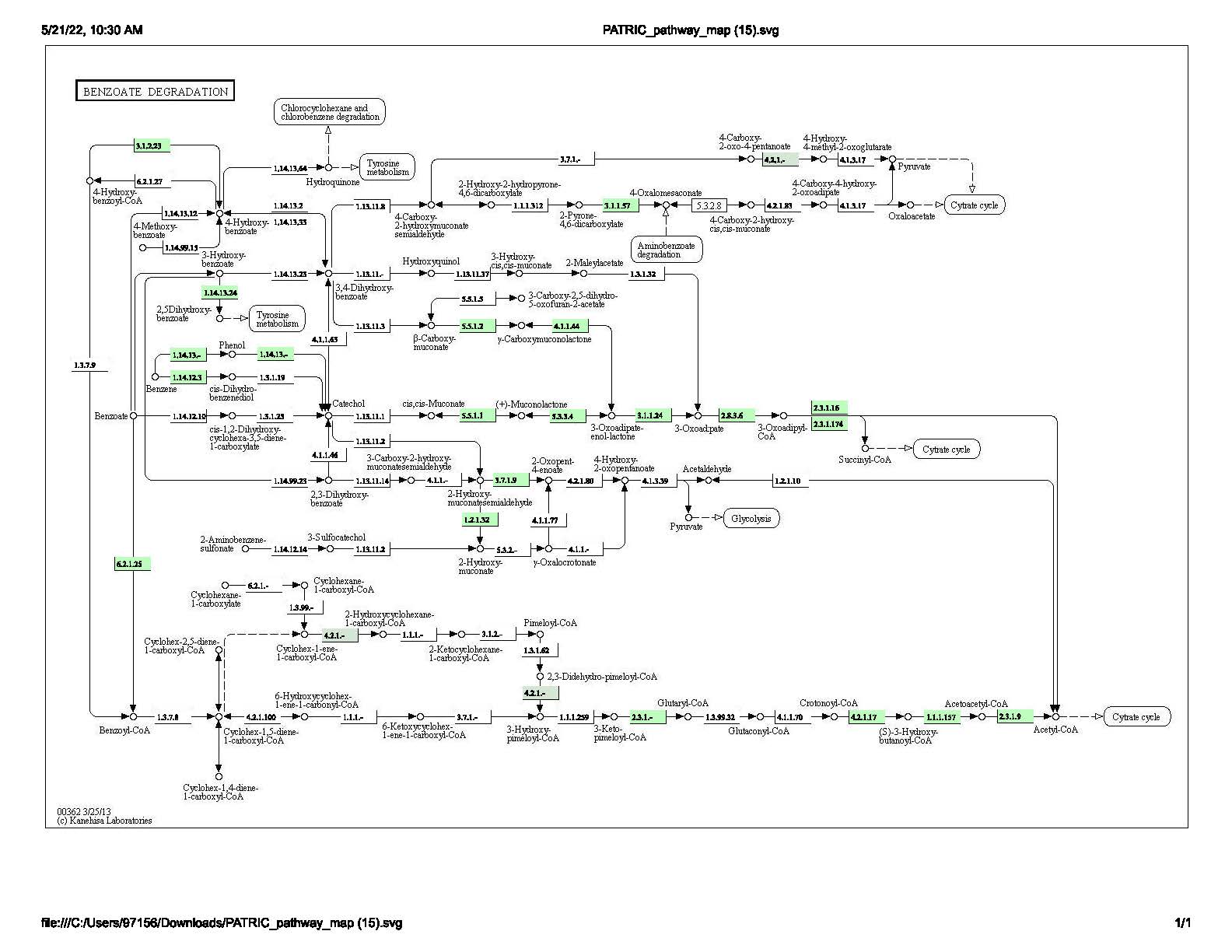


**Figure S15.** BNZ degradation pathway map00362 for A. x. C2 and A. x. KW38.


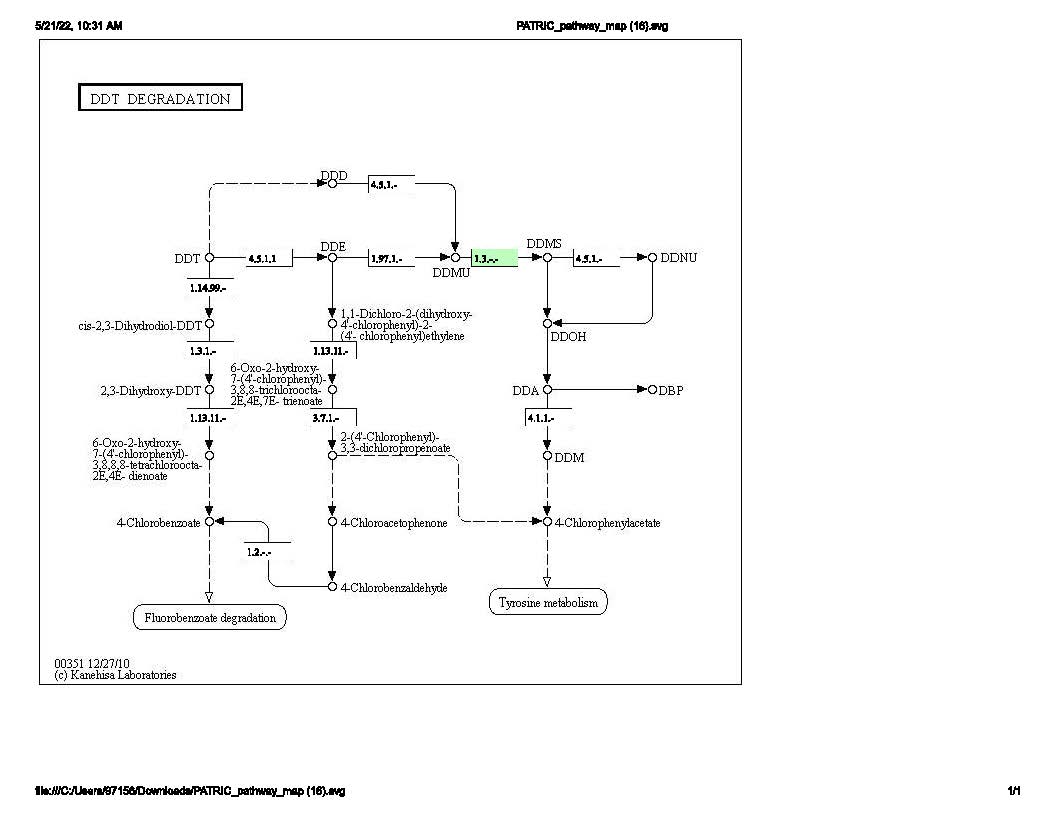


**Figure S16.** DDT degradation pathway map00351 for A. x. C2 and A. x. KW38.

**S-5. Selected genomes and associated metadata**

**Table S1.** The list of genomes in the selected study group for comparative analysis including UAE indigenous strains and the six other closest relatives from the phylogenetic tree analysis.

| Genome Name | Origin | Source | Length (Mbp^[[1]](#footnote-2)^) | Contig | Status |
| --- | --- | --- | --- | --- | --- |
| *A. xylosoxidans* C2 | UAE | Rhizosphere of the desert plants | 6.542959 | 1 | Private |
| *A. xylosoxidans* KW38 | UAE | Oil-contaminated water sample | 6.537038 | 1 | Private |
| *A. arsenitoxydans* SY8 | NA^[[2]](#footnote-3)^ | Soil of a pig farm with arsenic contamination | 6.156664 | 105 | WGS^[[3]](#footnote-4)^ |
| *A.* *piechaudii* ATCC 43553 | France | Nose wound | 6.154438 | 26 | WGS |
| *A.* sp. DMS1 | India | Industrial effluent contaminated site | 4.941236 | 356 | WGS |
| *A.xylosoxidans* A8 | NA | soil contaminated with (PCBs^[[4]](#footnote-5)^) | 7.359146 | 3 | Complete |
| *A.* *xylosoxidans* strain NCTC10807 | Japan | Human | 6.813182 | 1 | Complete |
| *A. denitrificans* NBRC 15125 | NA | NA | 6.700253 | 98 | WGS |

**S-6. Aromatic Hydrocarbon Degradation Pathways and Associated Enzymes List of pathways and enzymes responsible for degradation of aromatic hydrocarbons in the study group**

**Table S2.** List of pathways available in the selected genome group, the selected pathways are highlighted in grey[1].

| Pathway ID | Pathway Name | Pathway Class |
| --- | --- | --- |
| 280 | Valine, Leucine and isoleucine degradation | Amino Acid Metabolism |
| 270 | cysteine and methionine metabolism | Amino Acid Metabolism |
| 360 | Phenylalanine metabolism | Amino Acid Metabolism |
| 380 | Tryptophane metabolism | Amino Acid Metabolism |
| 300 | Lysine biosynthesis | Amino Acid Metabolism |
| 340 | Histidine metabolism | Amino Acid Metabolism |
| 310 | Lysine degradation | Amino Acid Metabolism |
| 250 | Alanine, aspartate and glutamate metabolism | Amino Acid Metabolism |
| 330 | Arginine and proline metabolism | Amino Acid Metabolism |
| 400 | Phenylalanine, tyrosine and tryptophan biosynthesis | Amino Acid Metabolism |
| 290 | Valine, Leucine and isoleucine degradation | Amino Acid Metabolism |
| 522 | Biosynthesis of 12-,14- and 16-membered macrolides | Biosynthesis of Polyketides and Nonribosomal Peptides |
| 523 | Polyketide sugar unit biosynthesis | Biosynthesis of Polyketides and Nonribosomal Peptides |
| 1051 | Biosynthesis of ansamycins | Biosynthesis of Polyketides and Nonribosomal Peptides |
| 1053 | Biosynthesis of siderophore group nonribosomal peptides | Biosynthesis of Polyketides and Nonribosomal Peptides |
| 1055 | Biosynthesis of vancomycin group antibiotics | Biosynthesis of Polyketides and Nonribosomal Peptides |
| 1056 | Biosynthesis of type II polyketide backbone | Biosynthesis of Polyketides and Nonribosomal Peptides |
| 1057 | Biosynthesis of type II polyketide products | Biosynthesis of Polyketides and Nonribosomal Peptides |
| 231 | Puromycin biosynthesis | Biosynthesis of Secondary Metabolites |
| 232 | Caffeine metabolism | Biosynthesis of Secondary Metabolites |
| 253 | Tetracycline biosynthesis | Biosynthesis of Secondary Metabolites |
| 311 | Penicillin and cephalosporin biosynthesis | Biosynthesis of Secondary Metabolites |
| 312 | *beta*-Lactam resistance | Biosynthesis of Secondary Metabolites |
| 401 | Novobiocin biosynthesis | Biosynthesis of Secondary Metabolites |
| 521 | Streptomycin biosynthesis | Biosynthesis of Secondary Metabolites |
| 900 | Terpenoid backbone biosynthesis | Biosynthesis of Secondary Metabolites |
| 903 | Limonene and pinene degradation | Biosynthesis of Secondary Metabolites |
| 904 | Diterpenoid biosynthesis | Biosynthesis of Secondary Metabolites |
| 905 | Brassinosteroid biosynthesis | Biosynthesis of Secondary Metabolites |
| 906 | Carotenoid biosynthesis | Biosynthesis of Secondary Metabolites |
| 908 | Zeatin biosynthesis | Biosynthesis of Secondary Metabolites |
| 909 | Sesquiterpenoid biosynthesis | Biosynthesis of Secondary Metabolites |
| 940 | Phenylpropanoid biosynthesis | Biosynthesis of Secondary Metabolites |
| 941 | Flavonoid biosynthesis | Biosynthesis of Secondary Metabolites |
| 942 | Anthocyanin biosynthesis | Biosynthesis of Secondary Metabolites |
| 943 | Isoflavonoid biosynthesis | Biosynthesis of Secondary Metabolites |
| 944 | Flavone and flavonol biosynthesis | Biosynthesis of Secondary Metabolites |
| 945 | Stilbenoid, diarylheptanoid and gingerol biosynthesis | Biosynthesis of Secondary Metabolites |
| 950 | Isoquinoline alkaloid biosynthesis | Biosynthesis of Secondary Metabolites |
| 960 | Tropane, piperidine and pyridine alkaloid biosynthesis | Biosynthesis of Secondary Metabolites |
| 965 | Betalain biosynthesis | Biosynthesis of Secondary Metabolites |
| 981 | Insect hormone biosynthesis | Biosynthesis of Secondary Metabolites |
| 10 | Glycolysis/ Gluconeogenesis | Carbohydrate Metabolism |
| 20 | Citrate cycle (TCA cycle) | Carbohydrate Metabolism |
| 30 | Pentose phosphate pathway | Carbohydrate Metabolism |
| 40 | Pentose and glucuronate interconversions | Carbohydrate Metabolism |
| 51 | Fructose and mannose metabolism | Carbohydrate Metabolism |
| 52 | Galactose metabolism | Carbohydrate Metabolism |
| 53 | Ascorbate and aldarate metabolism | Carbohydrate Metabolism |
| 500 | Starch and sucrose metabolism | Carbohydrate Metabolism |
| 520 | Amino sugar and nucleotide sugar metabolism | Carbohydrate Metabolism |
| 562 | Inositol phosphate metabolism | Carbohydrate Metabolism |
| 620 | Pyruvate metabolism | Carbohydrate Metabolism |
| 630 | Glyoxylate and dicarboxylate metabolism | Carbohydrate Metabolism |
| 640 | Propanoate metabolism | Carbohydrate Metabolism |
| 650 | Butanoate metabolism | Carbohydrate Metabolism |
| 660 | C5-Branched dibasic acid metabolism | Carbohydrate Metabolism |
| 190 | Oxidative phosphorylation | Energy Metabolism |
| 195 | Photosynthesis | Energy Metabolism |
| 680 | Methane metabolism | Energy Metabolism |
| 710 | Carbon fixation in photosynthetic organisms | Energy Metabolism |
| 720 | Reductive carboxylate cycle (CO_2_ fixation) | Energy Metabolism |
| 910 | Nitrogen metabolism | Energy Metabolism |
| 920 | Sulfur metabolism | Energy Metabolism |
| 510 | N-Glycan biosynthesis | Glycan Biosynthesis and Metabolism |
| 512 | O-Glycan biosynthesis | Glycan Biosynthesis and Metabolism |
| 513 | High-mannose type N-glycan biosynthesis | Glycan Biosynthesis and Metabolism |
| 531 | Glycosaminoglycan degradation | Glycan Biosynthesis and Metabolism |
| 540 | Lipopolysaccharide biosynthesis | Glycan Biosynthesis and Metabolism |
| 550 | Peptidoglycan biosynthesis | Glycan Biosynthesis and Metabolism |
| 563 | Glycosylphosphatidylinositol(GPI)-anchor biosynthesis | Glycan Biosynthesis and Metabolism |
| 601 | Glycosphingolipid biosynthesis - lacto and neolacto series | Glycan Biosynthesis and Metabolism |
| 603 | Glycosphingolipid biosynthesis - globo series | Glycan Biosynthesis and Metabolism |
| 604 | Glycosphingolipid biosynthesis - ganglio series | Glycan Biosynthesis and Metabolism |
| 4660 | T cell receptor signaling pathway | Immune System |
| 61 | Fatty acid biosynthesis | Lipid Metabolism |
| 62 | Fatty acid elongation in mitochondria | Lipid Metabolism |
| 71 | Fatty acid metabolism | Lipid Metabolism |
| 72 | Synthesis and degradation of ketone bodies | Lipid Metabolism |
| 100 | Steroid biosynthesis | Lipid Metabolism |
| 120 | Primary bile acid biosynthesis | Lipid Metabolism |
| 121 | Secondary bile acid biosynthesis | Lipid Metabolism |
| 140 | C21-Steroid hormone metabolism | Lipid Metabolism |
| 561 | Glycerolipid metabolism | Lipid Metabolism |
| 564 | Glycerophospholipid metabolism | Lipid Metabolism |
| 565 | Ether lipid metabolism | Lipid Metabolism |
| 590 | Arachidonic acid metabolism | Lipid Metabolism |
| 591 | Linoleic acid metabolism | Lipid Metabolism |
| 592 | *alpha*-Linolenic acid metabolism | Lipid Metabolism |
| 600 | Sphingolipid metabolism | Lipid Metabolism |
| 1040 | Biosynthesis of unsaturated fatty acids | Lipid Metabolism |
| 130 | Ubiquinone and other terpenoid-quinone biosynthesis | Metabolism of Cofactors and Vitamins |
| 670 | One carbon pool by folate | Metabolism of Cofactors and Vitamins |
| 730 | Thiamine metabolism | Metabolism of Cofactors and Vitamins |
| 740 | Riboflavin metabolism | Metabolism of Cofactors and Vitamins |
| 750 | Vitamin B6 metabolism | Metabolism of Cofactors and Vitamins |
| 760 | Nicotinate and nicotinamide metabolism | Metabolism of Cofactors and Vitamins |
| 770 | Pantothenate and CoA biosynthesis | Metabolism of Cofactors and Vitamins |
| 780 | Biotin metabolism | Metabolism of Cofactors and Vitamins |
| 785 | Lipoic acid metabolism | Metabolism of Cofactors and Vitamins |
| 790 | Folate biosynthesis | Metabolism of Cofactors and Vitamins |
| 830 | Retinol metabolism | Metabolism of Cofactors and Vitamins |
| 860 | Porphyrin and chlorophyll metabolism | Metabolism of Cofactors and Vitamins |
| 410 | *beta*-Alanine metabolism | Metabolism of Other Amino Acids |
| 430 | Taurine and hypotaurine metabolism | Metabolism of Other Amino Acids |
| 440 | Phosphonate and phosphinate metabolism | Metabolism of Other Amino Acids |
| 450 | Selenoamino acid metabolism | Metabolism of Other Amino Acids |
| 460 | Cyanoamino acid metabolism | Metabolism of Other Amino Acids |
| 471 | D-Glutamine and D-glutamate metabolism | Metabolism of Other Amino Acids |
| 472 | D-Arginine and D-ornithine metabolism | Metabolism of Other Amino Acids |
| 473 | D-Alanine metabolism | Metabolism of Other Amino Acids |
| 480 | Glutathione metabolism | Metabolism of Other Amino Acids |
| 230 | Purine metabolism | Nucleotide Metabolism |
| 240 | Pyrimidine metabolism | Nucleotide Metabolism |
| 4070 | Phosphatidylinositol signaling system | Signal Transduction |
| 4150 | mTOR signaling pathway | Signal Transduction |
| 970 | Aminoacyl-tRNA biosynthesis | Translation |
| 281 | Geraniol degradation | Xenobiotics Biodegradation and Metabolism |
| 351 | 1,1,1-Trichloro-2,2-bis(4-chlorophenyl)ethane (DDT) degradation | Xenobiotics Biodegradation and Metabolism |
| 361 | gamma-Hexachlorocyclohexane degradation | Xenobiotics Biodegradation and Metabolism |
| 362 | Benzoate degradation via hydroxylation | Xenobiotics Biodegradation and Metabolism |
| 363 | Bisphenol A degradation | Xenobiotics Biodegradation and Metabolism |
| 364 | Fluorobenzoate degradation | Xenobiotics Biodegradation and Metabolism |
| 621 | Biphenyl degradation | Xenobiotics Biodegradation and Metabolism |
| 622 | Toluene and xylene degradation | Xenobiotics Biodegradation and Metabolism |
| 623 | 2,4-Dichlorobenzoate degradation | Xenobiotics Biodegradation and Metabolism |
| 624 | 1- and 2-Methylnaphthalene degradation | Xenobiotics Biodegradation and Metabolism |
| 625 | Tetrachloroethene degradation | Xenobiotics Biodegradation and Metabolism |
| 626 | Naphthalene and anthracene degradation | Xenobiotics Biodegradation and Metabolism |
| 627 | 1,4-Dichlorobenzene degradation | Xenobiotics Biodegradation and Metabolism |
| 633 | Trinitrotoluene degradation | Xenobiotics Biodegradation and Metabolism |
| 642 | Ethylbenzene degradation | Xenobiotics Biodegradation and Metabolism |
| 643 | Styrene degradation | Xenobiotics Biodegradation and Metabolism |
| 791 | Atrazine degradation | Xenobiotics Biodegradation and Metabolism |
| 930 | Caprolactam degradation | Xenobiotics Biodegradation and Metabolism |
| 980 | Metabolism of xenobiotics by cytochrome P450 | Xenobiotics Biodegradation and Metabolism |
| 982 | Drug metabolism - cytochrome P450 | Xenobiotics Biodegradation and Metabolism |
| 983 | Drug metabolism - other enzymes | Xenobiotics Biodegradation and Metabolism |
| 523 | Polyketide sugar unit biosynthesis | Biosynthesis of Polyketides and Nonribosomal Peptides |
| 1051 | Biosynthesis of ansamycins | Biosynthesis of Polyketides and Nonribosomal Peptides |

**Table S3.** The list of enzymes involved in the degradation of aromatic structures available in the selected study group. In grey are the enzymes that are not available in A. x. C2 and A. x. KW38 (which are not threaded into 3D structures in the supplementary document). In green is the enzyme absent in A. x. KW38. In blue, is the enzyme found only in A. x. C2 and A. x. KW38.

| # | Enzyme EC [1], [2] | Symbol [2] | BV-BRC Enzyme Name [1] | KEGG Enzyme Name [2] | | Pathways [2] |
| --- | --- | --- | --- | --- | --- | --- |
| 1 | 1.1.1.- | *badH* | With NAD(+) or NADP(+) as acceptor | 2-hydroxycyclohexanecarboxylic-*CoA* dehydrogenase | | ko00362, map00363 |
| 2 | 1.1.1.1 | *adhP* | alcohol dehydrogenase | alcohol dehydrogenase, propanol-preferring | | map00626 |
| 3 | 1.1.1.157 | *paaH, hbd, fadB, mmgB* | 3-hydroxybutyryl-CoA dehydrogenase | | | ko00362 |
| 4 | 1.13.11.1 | *catA* | Salicylate 1-monooxygenase | catechol 1,2-dioxygenase | | ko00362 |
| 5 | 1.13.11.3 | *pcaG* | protocatechuate 3,4-dioxygenase | protocatechuate 3,4-dioxygenase, alpha subunit | | ko00362, map00624 |
| 6 | 1.14.12.3 | *todC1, bedC1, tcbAa* | Benzene 1,2-dioxygenase | benzene/toluene/chlorobenzene dioxygenase subunit alpha | | ko00362 |
| 7 | 1.14.13.- | *-* | With NADH or NADPH as one donor, and incorporation of one atom of oxygen | phenanthrene 9,10-monooxygenase/ phenanthrene 1,2-monooxygenase | | map00626, map00624, ko00362, map00363 |
| 8 | 1.14.13.24 | *nagX* | 3-hydroxybenzoate 6-monooxygenase | | | ko00362 |
| 9 | 1.14.99.- | *-* | Miscellaneous | naphthalene disulfonate 1,2-dioxygenase | | map00626, map00351 |
| 10 | 1.2.1.10 | *mhpF* | Acetaldehyde dehydrogenase (acetylating) | acetaldehyde dehydrogenase | | ko00362, map00621 |
| 11 | 1.2.1.32 | *dmpC, xylG, praB* | Aminomuconate-semialdehyde dehydrogenase | aminomuconate-semialdehyde/2-hydroxymuconate-6-semialdehyde dehydrogenase | | ko00362 |
| 12 | 1.3.-.- | *-* | Acting on the CH-CH group of donors | benzo[a]pyrene-trans-11,12-dihydrodiol dehydrogenase | | map00624, map00351, map00363 |
| 13 | 2.1.1.- | *-* | Methyltransferases | 1-hydroxypyrene methyltransferase | | map00624 |
| 14 | 2.3.1.- | *bnsA* | Transferring groups other than amino-acyl groups | naphthyl-2-oxo methyl-succinyl-CoA thiolase subunit | | map00626, ko00362 |
| 15 | 2.3.1.16 | *fadA, fadI* | Acetyl-CoA C-acyltransferase | | | ko00362 |
| 16 | 2.3.1.174 | *pcaF* | 3-oxodipyl-CoA thiolase | | | ko00362 |
| 17 | 2.3.1.9 | *ACAT, atoB* | Acetyl-CoA C-acetyltransferase | | | ko00362 |
| 18 | 2.8.3.6 | *pcaI* | 3-oxoadipate CoA-transferase | 3-oxoadipate CoA-transferase, alpha subunit | | ko00362 |
| 19 | 3.1.1.- | *-* | Carboxylic ester hydrolases | 4-hydroxyphenyl-4-hydroxybenzoate hydrolase | | map00363 |
| 20 | 3.1.1.2 | *-* | arylesterase | | | map00363 |
| 21 | 3.1.1.24 | *pcaD* | 3-oxodipate enol-lactonase | | | ko00362 |
| 22 | 3.1.1.57 | *ligI* | 2-pyrone-4,6-dicarboxylate lactonase | | | ko00362 |
| 23 | 3.1.2.23 | *E3.1.2.23* | 4-hydroxybenzoyl-CoA thioesterase | | | ko00362 |
| 24 | 3.7.1.- | *flnE* | In ketonic substances | 2-hydroxy-6-oxo-6-(2'-carboxyphenyl)-hexa-2,4-dienoate hydrolase | | ko00362, map00621, map00351 |
| 25 | 3.7.1.9 | *dmpD, xylF* | 2-hydroxymuconate-semialdehyde hydrolase | | | ko00362 |
| 26 | 4.1.1.- | *-* | Carboxy-layses | 2-hydroxyisophthalate decarboxylase | | map00351, ko00362, map00621, map00626 |
| 27 | 4.1.1.44 | *pcaC* | 4-carboxymuconolactone decarboxylase | | | ko00362 |
| 28 | 4.1.1.77 | *dmpH, xylI, nahK* | 2-oxo-3-hexenedioate decarboxylase | | | ko00362, map00621 |
| 29 | 4.1.3.17 | *ligK, galC* | 4-hydroxy-4-methyl-2-oxoglutarate aldolase | | | ko00362 |
| 30 | 4.1.3.39 | *mhpE* | 4-hydroxy 2-oxovalerate aldolase | | | ko00362, map00621 |
| 31 | 4.2.1.- | *bnsH* | Hydro-lyases | | naphthyl-2-hydroxymethylsuccinyl-CoA hydratase | ko00362, map00363, map00626 |
| 32 | 4.2.1.17 | *paaF, echA* | enoyl-CoA hydratase | | | ko00362 |
| 33 | 4.2.1.83 | *ligJ* | 4-oxalmesaconate hydratase | | | ko00362 |
| 34 | 5.3.3.4 | *catC* | muconolactone D-isomerase | | | ko00362 |
| 35 | 5.5.1.1 | *catB* | muconate cycloisomerase | | | ko00362 |
| 36 | 5.5.1.2 | *pcaB* | 3-carboxy-cis,cis-muconate cycloisomerase | | | ko00362 |
| 37 | 6.2.1.25 | *badA* | benzoate-CoA ligase | | | ko00362 |

**Table S4.** Details of the selected enzymes for this study and the sequence curation details for every enzyme in A. x. C2 and A. x. KW38 in reference to the reference genomes and with global set of enzymes in NCBI confirming the BLAST Identities, positives, and gaps.

| # | Enzyme EC | Curated Public Reference protein family | BLAST Identities | BLAST Positives | BLAST Gap | Accession Number[3] |
| --- | --- | --- | --- | --- | --- | --- |
| 1 | 1.1.1.1 | iron-containing alcohol dehydrogenase | 99% | 99% | 0% | WP_026382448.1 |
| 2 | 1.1.1.157 | 3-hydroxyacyl-CoA dehydrogenase family protein | 99% | 99% | 0% |  |
| 3 | 1.14.12.3 | aromatic-ring-hydroxylating dioxygenase subunit beta | 99% | 99% | 0% | cd00667 |
| 4 | 1.14.13.- | flavin reductase family protein | 99 | 99 | 0 | WP_241130244.1 |
| 5 | 1.14.13.1 | FAD-dependent monooxygenase, partial | 99 | 100 | 0 | WP_241117339.1, OFL33735.1, WP_238926451.1, WP_076467759.1, WP_198140035.1, WP_082395066.1 |
| 6 | 1.14.13.24 | 3-hydroxybenzoate 6-monooxygenase | 99 | 100 | 0 | WP_104413809.1 |
| 7 | 1.2.1.32 | Aminomuconate-semialdehyde dehydrogenase | 99 | 99 | 0 | WP_148317163.1, WP_020926372.1, WP_049054026.1, WP_148317163.1 |
| 9 | 1.3.-.- | Acting on the CH-CH group of donors | 100 | 100 | 0 | WP_006384749.1, WP_192579587.1, WP_241052406.1, WP_173000892.1, WP_180187534.1 |
| 10 | 2.1.1.- | Methyltransferases | 86 | 89 | 0 | WP_054442614.1, WP_024070481.1, WP_238926997.1, WP_054518153.1, WP_054446768.1, WP_006385774.1, WP_026383958.1, WP_241116646.1 |
| 11 | 2.3.1.- | Transferring groups other than amino-acyl groups | 99 | 99 | 0 | WP_241134739.1, WP_054513357.1, WP_049054061.1, WP_241071417.1, WP_202731871.1 |
| 12 | 2.3.1.9 | Acetyl-CoA C-acetyltransferase | 99 | 99 | 0 | WP_047991572.1, WP_026384200.1, WP_241122106.1 |
| 13 | 2.3.1.174 | 3-oxodipyl-CoA thiolase |  |  |  |  |
| 14 | 2.3.1.16 | Acetyl-CoA C-acyltransferase | 100 | 100 | 0 | WP_047991572.1, WP_026384200.1 |
| 15 | 2.8.3.6 | 3-oxoadipate CoA-transferase, alpha subunit | 99 | 99 | 0 | WP_026381997.1 |
| 16 | 3.1.1.- | 4-hydroxyphenyl-4-hydroxybenzoate hydrolase | 97 | 100 | 0 | WP_054504802.1, WP_054446911.1, WP_020925085.1 |
| 17 | 3.1.1.2 | arylesterase | 99 | 100 | 0 | KMJ88982.1, WP_238926154.1, AHC47119.1 |
| 18 | 3.1.1.24 | 3-oxodipate enol-lactonase | 100 | 100 | 100 | WP_006390033.1, WP_054482073.1, WP_053500880.1 |
| 19 | 3.1.1.57 | 2-pyrone-4,6-dicarboxylate lactonase | 99 | 100 | 0 | KAA5921399.1, WP_173361318.1, KMJ87502.1 |
| 20 | 3.1.2.23 | 4-hydroxybenzoyl-CoA thioesterase | 100 | 100 | 0 | WP_047991572.1, WP_026384200.1, WP_006392716.1 |
| 21 | 3.7.1.9 | 2-hydroxymuconate-semialdehyde hydrolase | 100 | 100 | 0 | CUJ56697.1, WP_054482020.1, WP_026382082.1, WP_054446369.1, WP_024070707.1 |
| 22 | 4.1.1.44 | 4-carboxymuconolactone decarboxylase | 99 | 100 | 0 | WP_241130809.1, WP_049051011.1, WP_241135832.1, WP_163108801.1, WP_241076995.1, WP_054443018.1 |
| 23 | 4.2.1.- | naphthyl-2-hydroxymethylsuccinyl-CoA hydratase | 100 | 100 | 0 | KOQ31149.1, WP_082391602.1, WP_241122241.1, WP_020925877.1, WP_081004503.1, WP_082455238.1 |
| 24 | 4.2.1.17 | enoyl-CoA hydratase | 99 | 99 | 0 | WP_006388863.1, WP_175131138.1, WP_049054154.1, WP_054416447.1 |
| 25 | 5.3.3.4 | muconolactone D-isomerase | 97 | 100 | 0 | WP_054419420.1, WP_129153839.1, WP_006390036.1, WP_020926098.1 |
| 26 | 5.5.1.1 | muconate cycloisomerase | 99 | 99 | 0 | WP_054472885.1, WP_020924870.1, WP_026382307.1 |
| 27 | 5.5.1.2 | 3-carboxy-cis,cis-muconate cycloisomerase | 99 | 99 | 0 | WP_110134722.1, WP_054439944.1, WP_107316445.1, WP_241072136.1, WP_241123365.1 |
| 28 | 6.2.1.25 | benzoate-CoA ligase | 98 | 98 | 0 | WP_112957895.1, WP_218911502.1, WP_054516096.1, WP_054481906.1 |

**S-7. Enzyme Heatmaps**


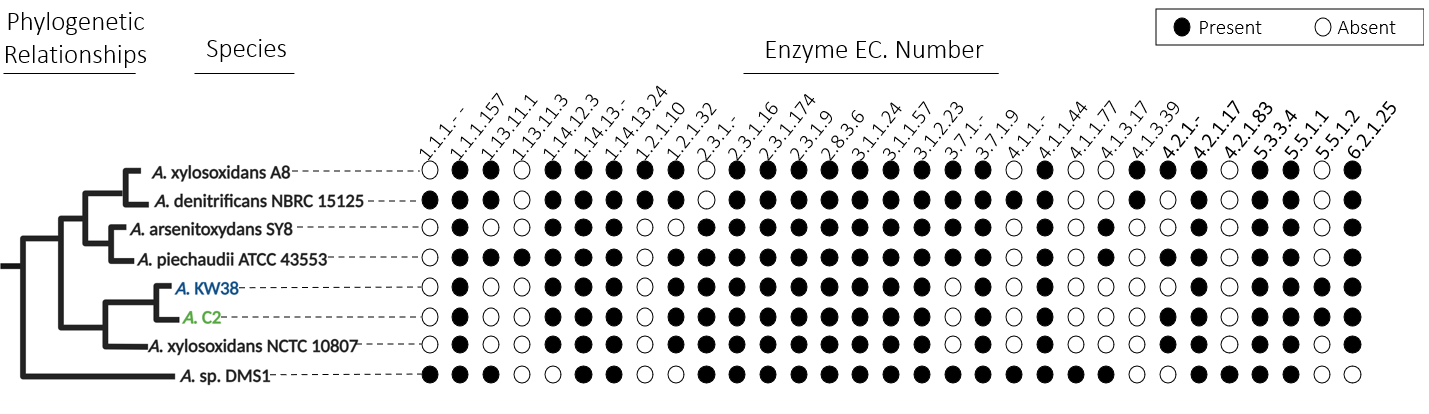


**Figure S17.** Distribution of enzymes related to BNZ degradation via hydroxylation pathway (map00362) in 8 genomes. Enzymes present or absent are represented by closed or open circles respectively.


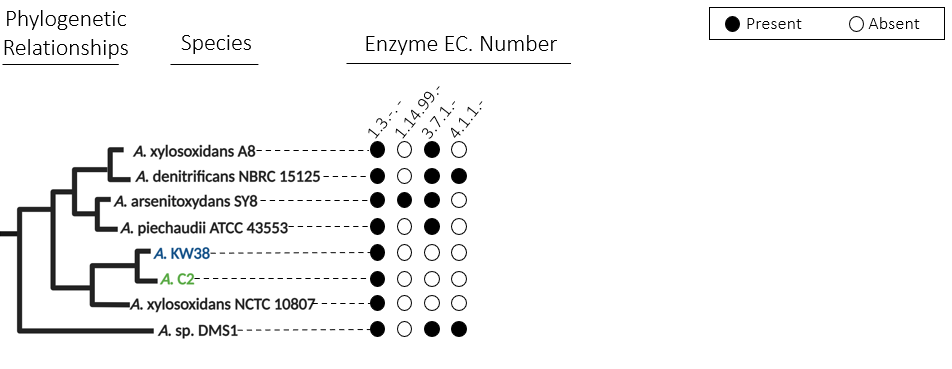


**Figure S18.** Distribution of enzymes related to 1,1,1-Trichloro-2,2-bis(4-chlorophenyl)ethane (DDT) degradation (map00351) in 8 genomes. Enzymes present or absent are represented by closed or open circles respectively.


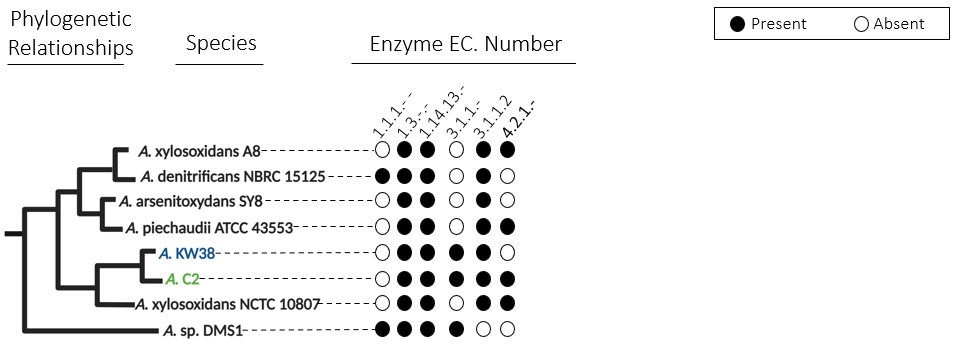


**Figure S19.** Distribution of enzymes related to BPA degradation (map00363) in 8 genomes. Enzymes present or absent are represented by closed or open circles respectively.


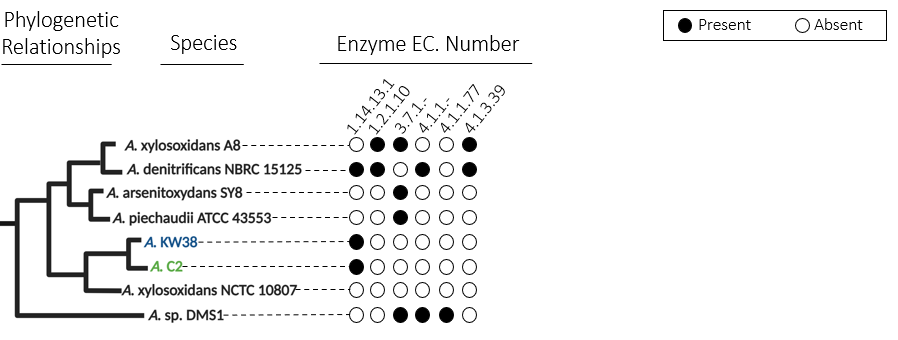


**Figure S20.** Distribution of enzymes related to B degradation (map00621) in 8 genomes. Enzymes present or absent are represented by closed or open circles respectively.


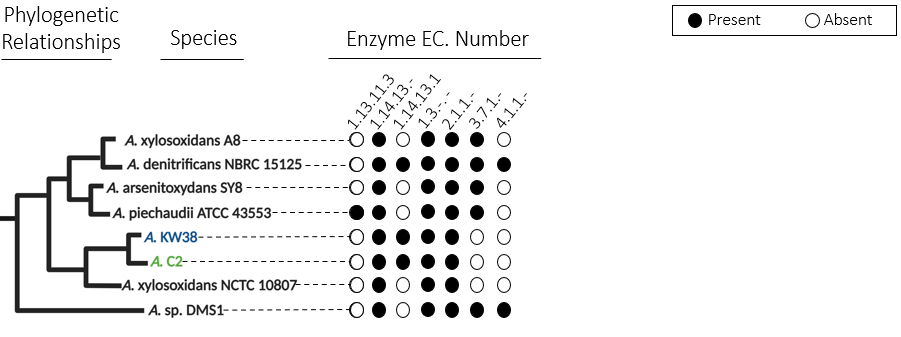


**Figure S21.** Distribution of enzymes related to MN degradation (map00624) in 8 genomes. Enzymes present or absent are represented by closed or open circles respectively.


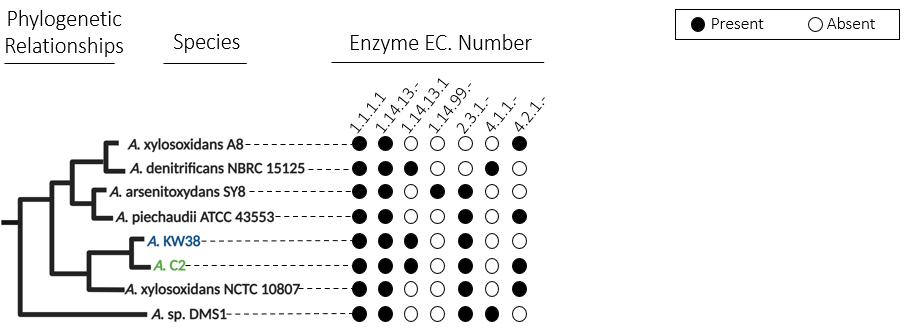


**Figure S22.** Distribution of enzymes related to NAP and ANT degradation (map00626) in 8 genomes. Enzymes present or absent are represented by closed or open circles respectively.

**S-8. Threaded enzyme structures**


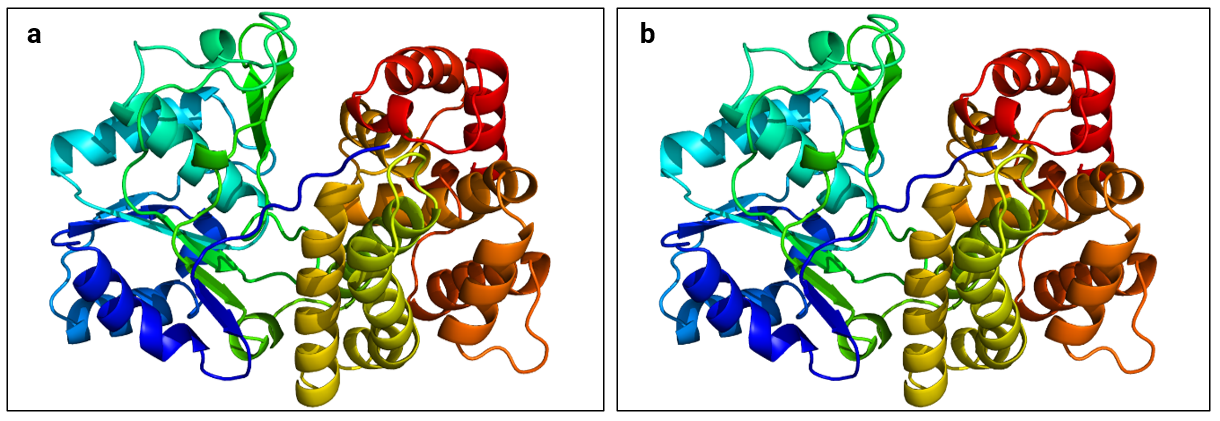


**Figure S23.** Visualization of the final predicted structure of enzyme (EC.1.1.1.1) in **a**. A. x. C2 and **b**. A. x. KW38.


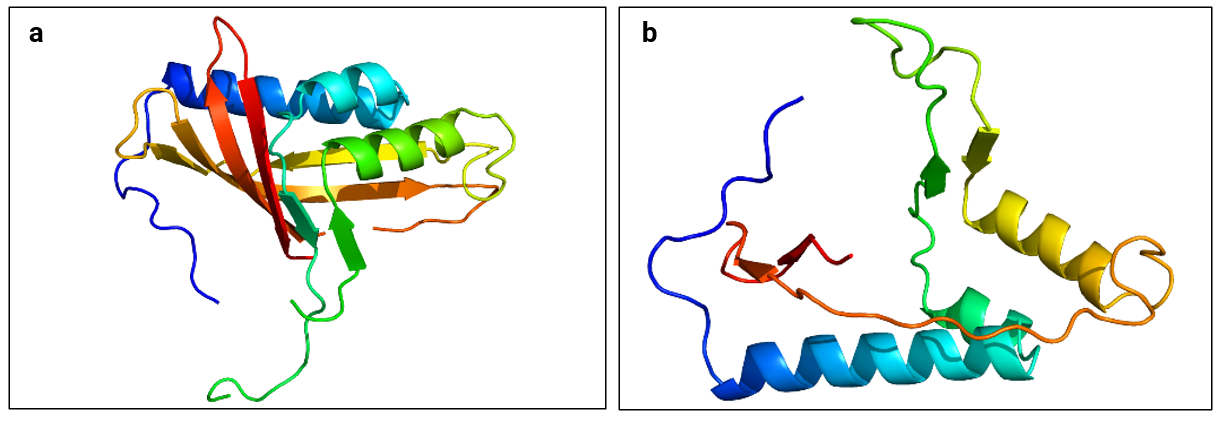


**Figure S24.** Visualization of the final predicted structure of enzyme (EC.1.14.12.3) in **a**. A. x. C2 and **b**. A. x. KW38.


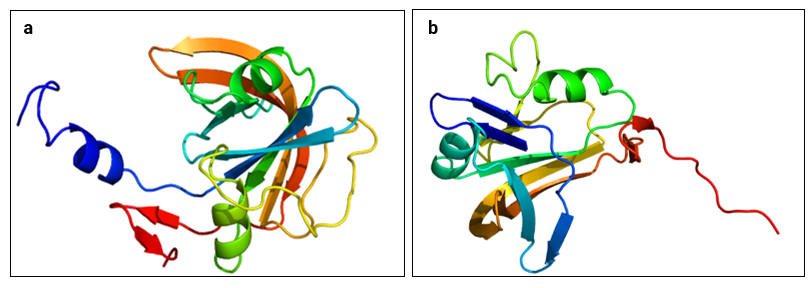


**Figure S25.** Visualization of the final predicted structure of enzyme (EC.1.14.13.-) in **a**. A. x. C2 and **b**. A. x. KW38.


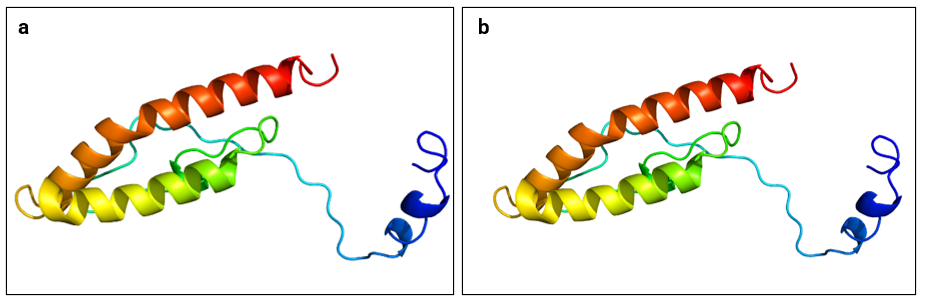


**Figure S26.** Visualization of the final predicted structure of enzyme (EC. 1.14.13.1) in **a***. A. x.* C2 *and* ***b****. A. x*. KW38.
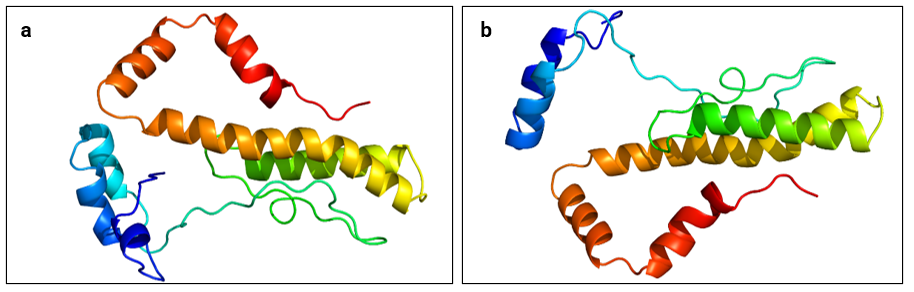


**Figure S27.** Visualization of the final predicted structure of enzyme (EC. 1.14.13.24) in **a**. A. x. C2 and **b**. A. x. KW38.


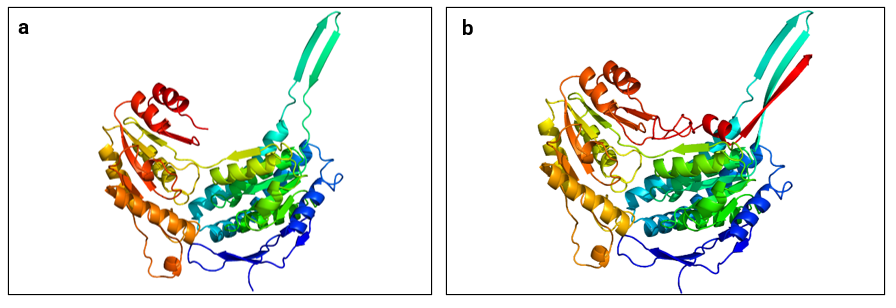


**Figure S28.** Visualization of the final predicted structure of enzyme (EC.1.2.1.32) in **a**. A. x. C2 and **b**. A. x. KW38.


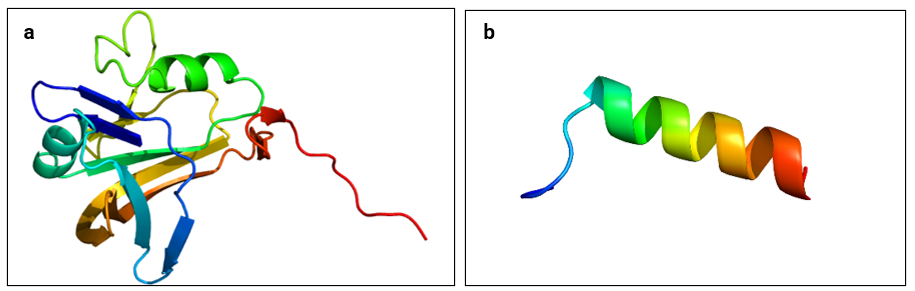


**Figure S29.** Visualization of the final predicted structure of enzyme (EC.1.3.-.-) in **a**. A. x. C2 and **b**. A. x. KW38.


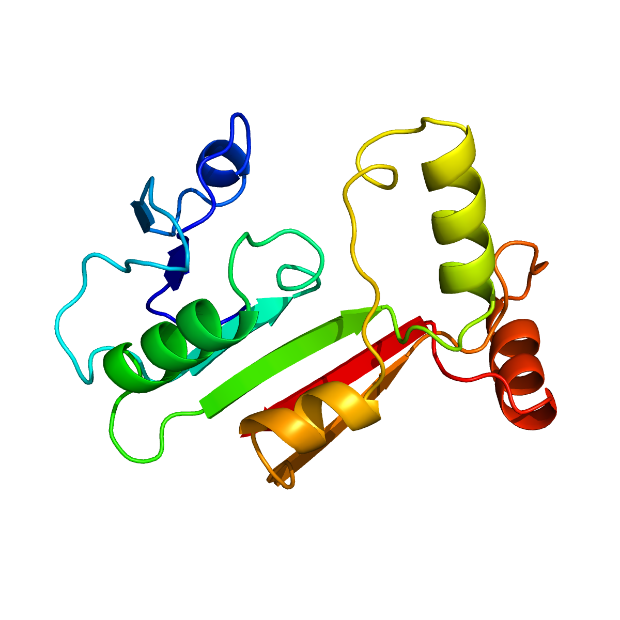


**Figure S30.** Visualization of the final predicted structure of enzyme (EC.2.1.1.-) in A. x. KW38.


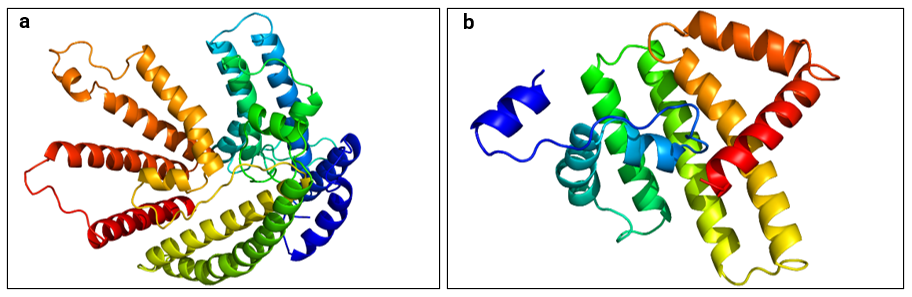


**Figure S31.** Visualization of the final predicted structure of enzyme (EC.2.3.1.-) in **a**. A. x. C2 and **b**. A. x. KW38.


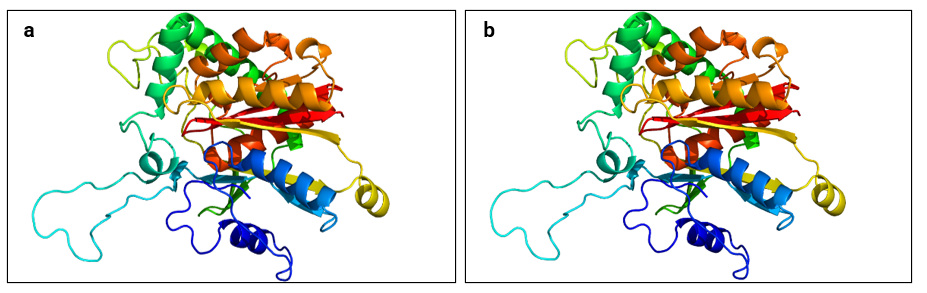


**Figure S32.** Visualization of the final predicted structure of enzyme (EC.2.3.1.16) in **a**. A. x. C2 and **b**. A. x. KW38.


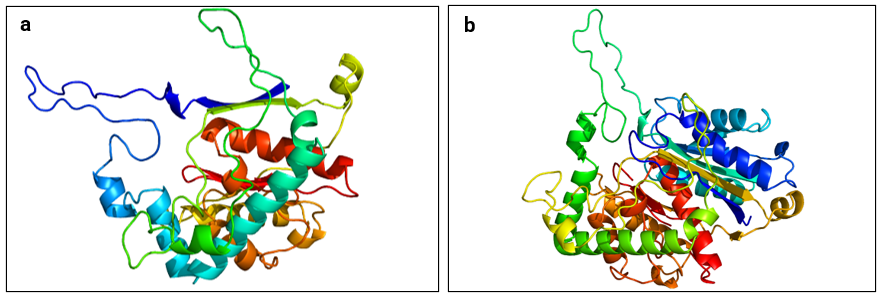


**Figure S33.** Visualization of the final predicted structure of enzyme (EC.2.3.1.174) in **a**. A. x. C2 and **b**. A. x. KW38.


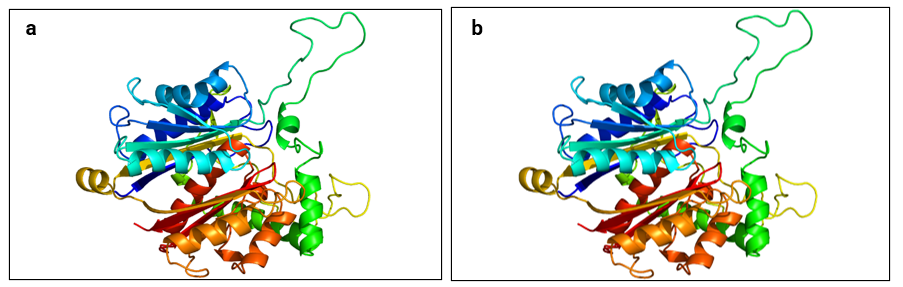


**Figure S34.** Visualization of the final predicted structure of enzyme (EC.2.3.1.9) in **a**. A. x. C2 and **b**. A. x. KW38.


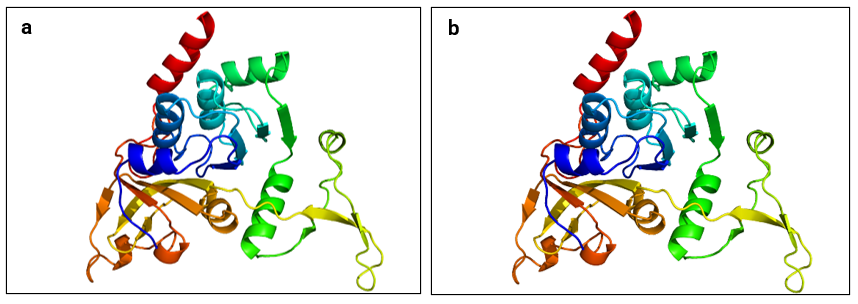


**Figure S35.** Visualization of the final predicted structure of enzyme (EC.2.8.3.6) in **a**. A. x. C2 and **b**. A. x. KW38.


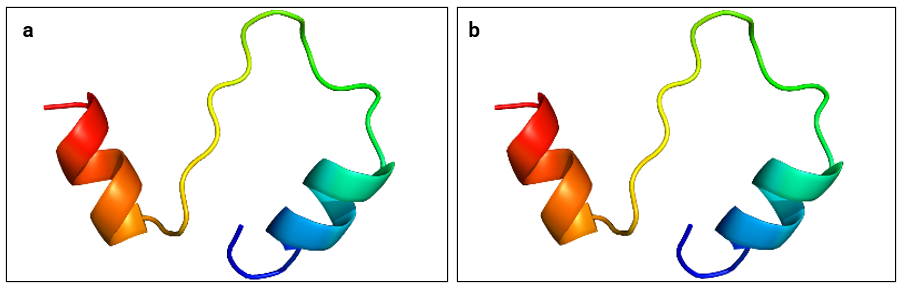


**Figure S36.** Visualization of the final predicted structure of enzyme (EC.3.1.1.-) in **a**. A. x. C2 and **b**. A. x. KW38.


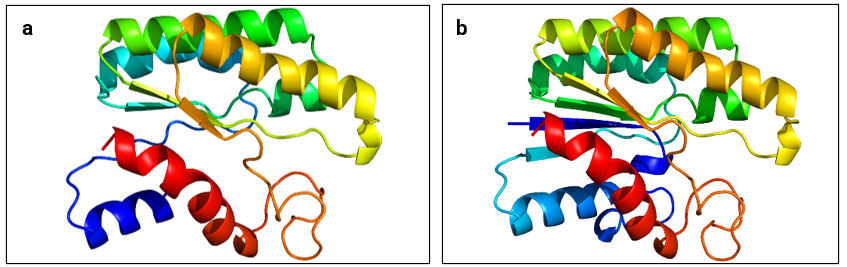


**Figure S37.** Visualization of the final predicted structure of enzyme (EC.3.1.1.2) in **a**. A. x.C2 and **b**. A. x. KW38.


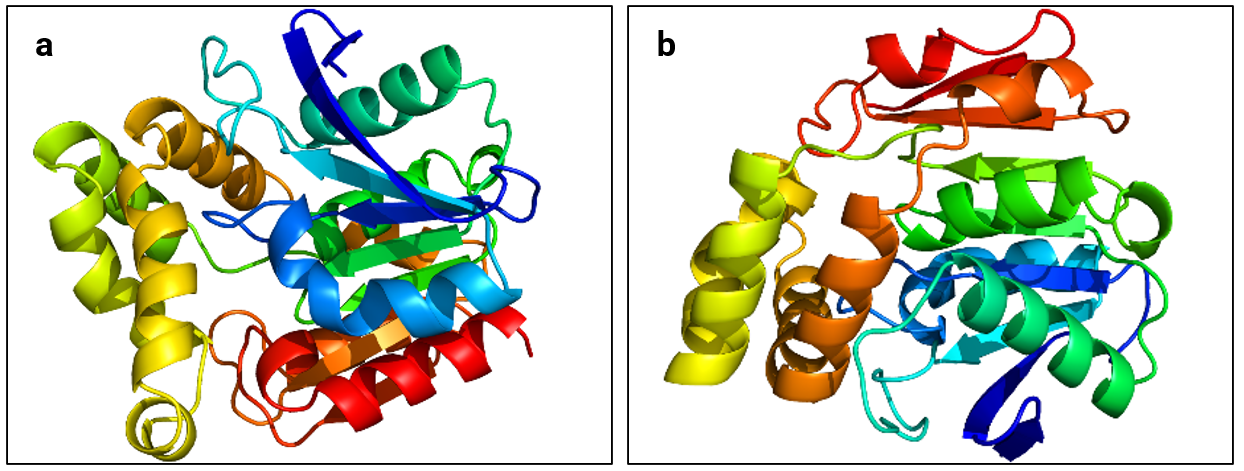


**Figure S38.** Visualization of the final predicted structure of enzyme (EC.3.1.1.24) in **a**. A. x. C2 and **b**. A. x. KW38.


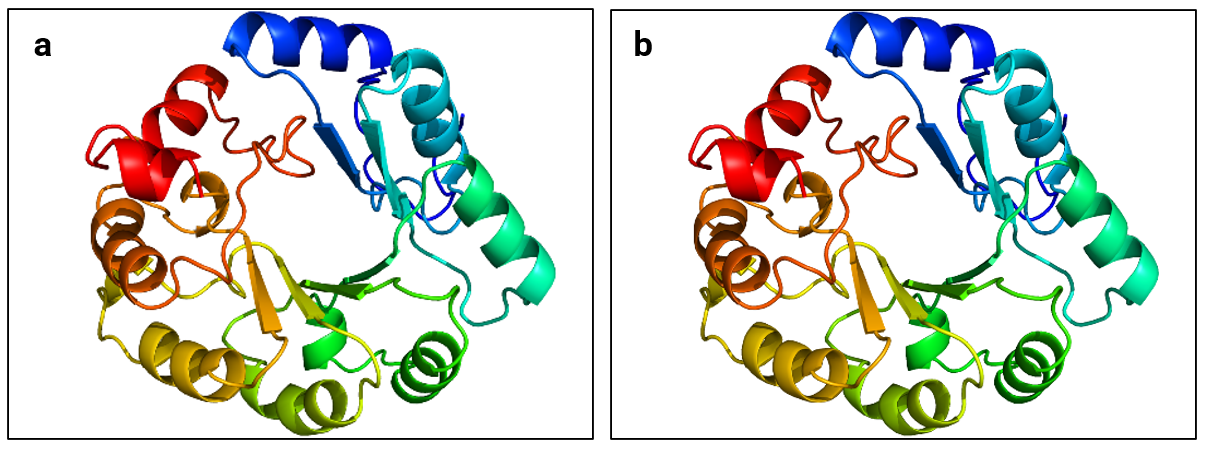


**Figure S39.** Visualization of the final predicted structure of enzyme (EC.3.1.1.57) in **a**. A. x. C2 and **b**. A. x. KW38.


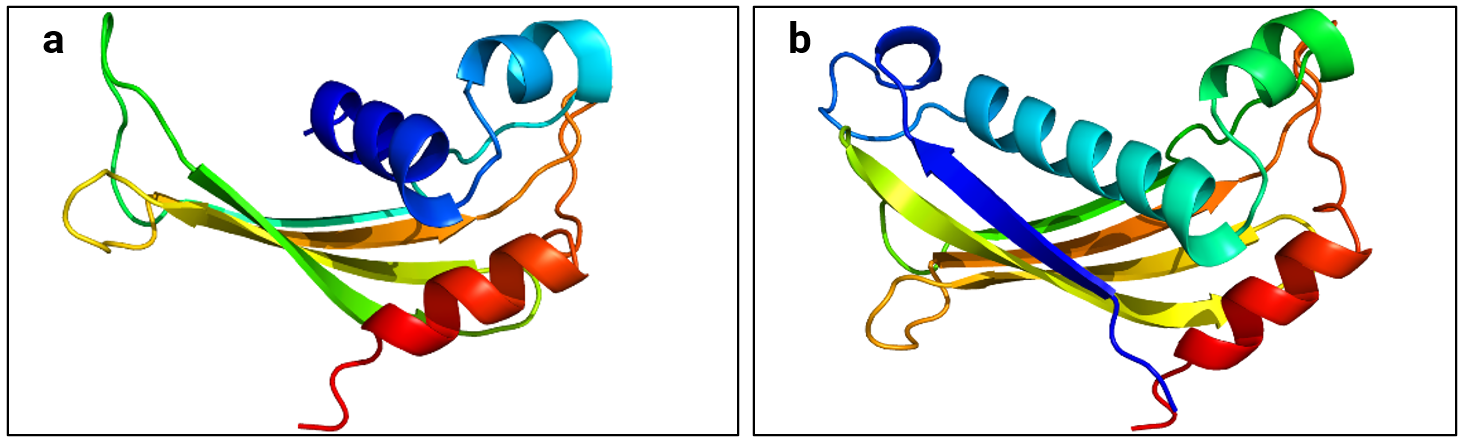


**Figure S40.** Visualization of the final predicted structure of enzyme (EC.3.1.2.23) in **a**. A. x. C2 and **b**. A. x. KW38.


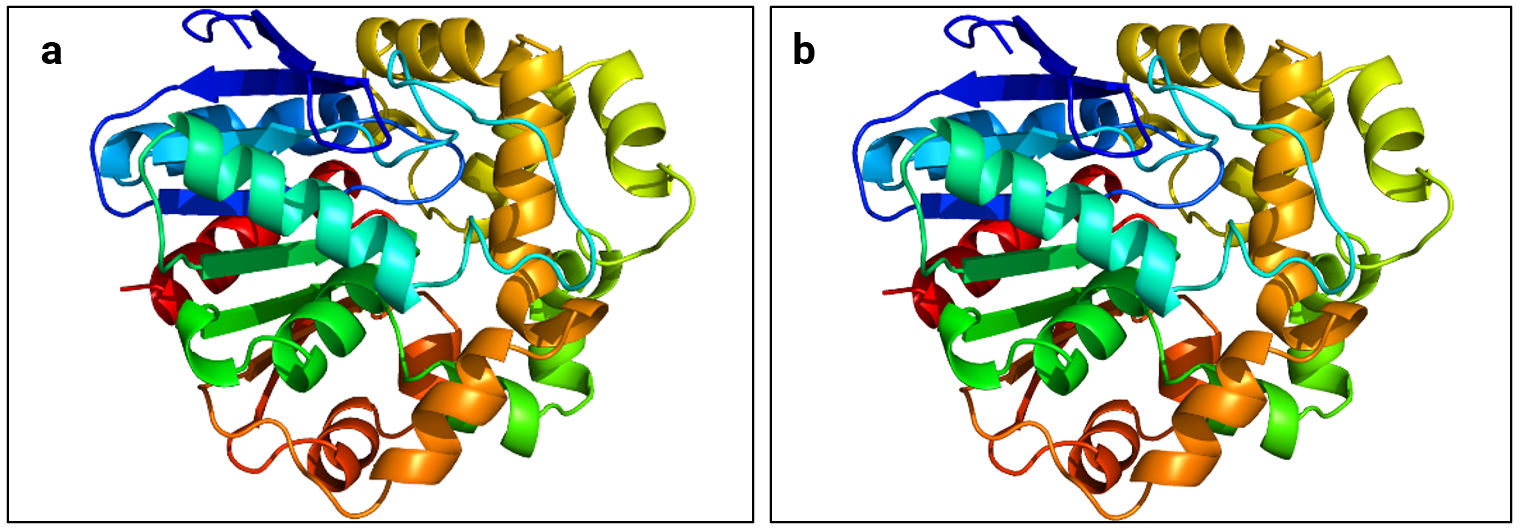


**Figure S41.** Visualization of the final predicted structure of enzyme (EC.3.7.1.9) in **a**. A. x. C2 and **b**. A. x. KW38.


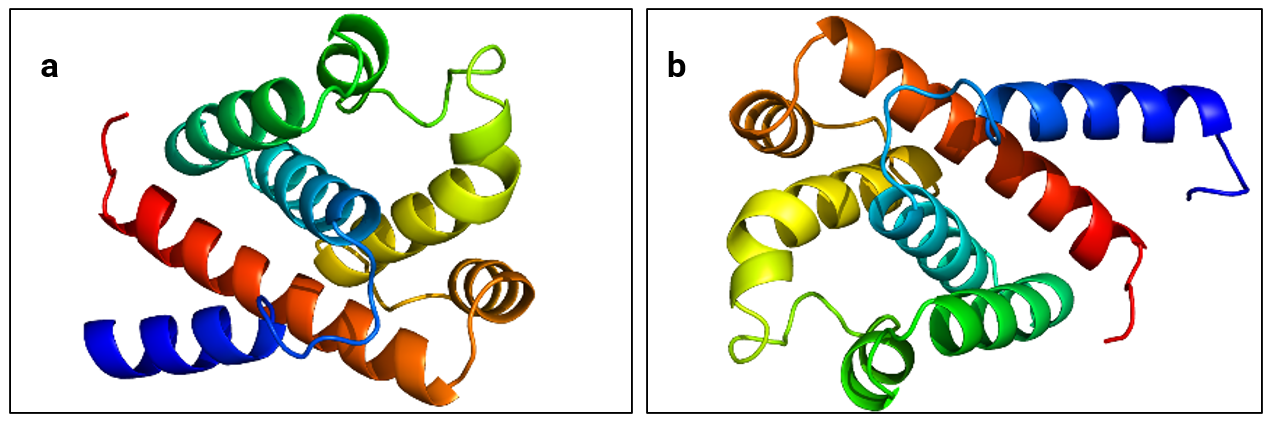


**Figure S42.** Visualization of the final predicted structure of enzyme (EC.4.1.1.44) in **a**. A. x. C2 and **b**. A. x. KW38.


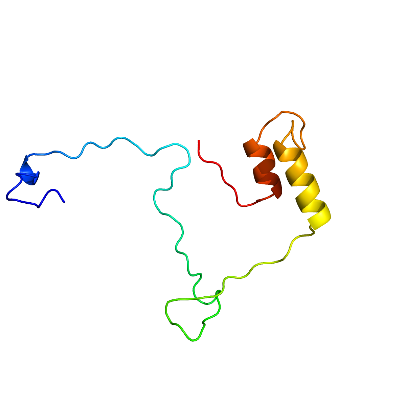


**Figure S43.** Visualization of the final predicted structure of enzyme (EC.4.2.1.-) in A. x. C2.


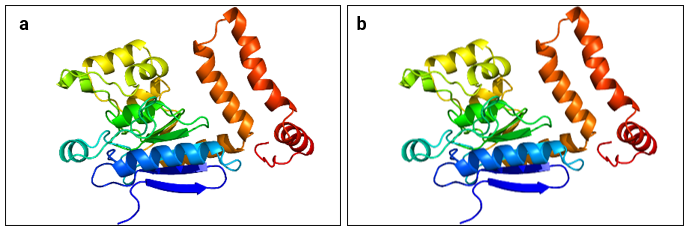


**Figure S44.** Visualization of the final predicted structure of enzyme (EC.4.2.1.17) in **a**. A. x. C2 and **b**. A. x. KW38.


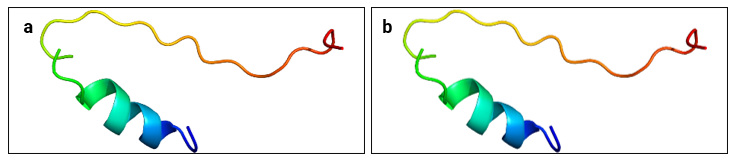


**Figure S45.** Visualization of the final predicted structure of enzyme (EC. 5.3.3.4) in **a**. A. x. C2 and **b**. A. x. KW38.


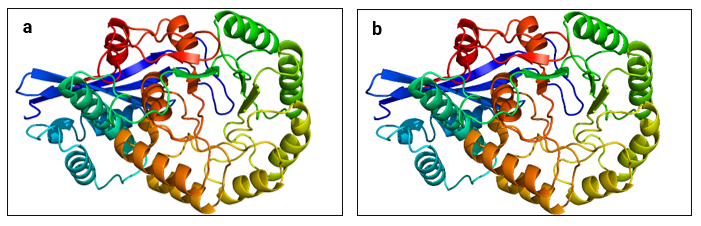


**Figure S46.** Visualization of the final predicted structure of enzyme (EC. 5.5.1.1) in **a**. A. x. C2 and **b**. A. x. KW38.


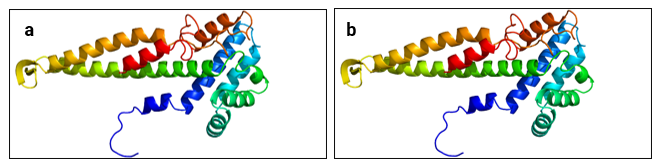


**Figure S47.** Visualization of the final predicted structure of enzyme (EC.5.5.1.2) in **a**. A. x. C2 and **b**. A. x. KW38.


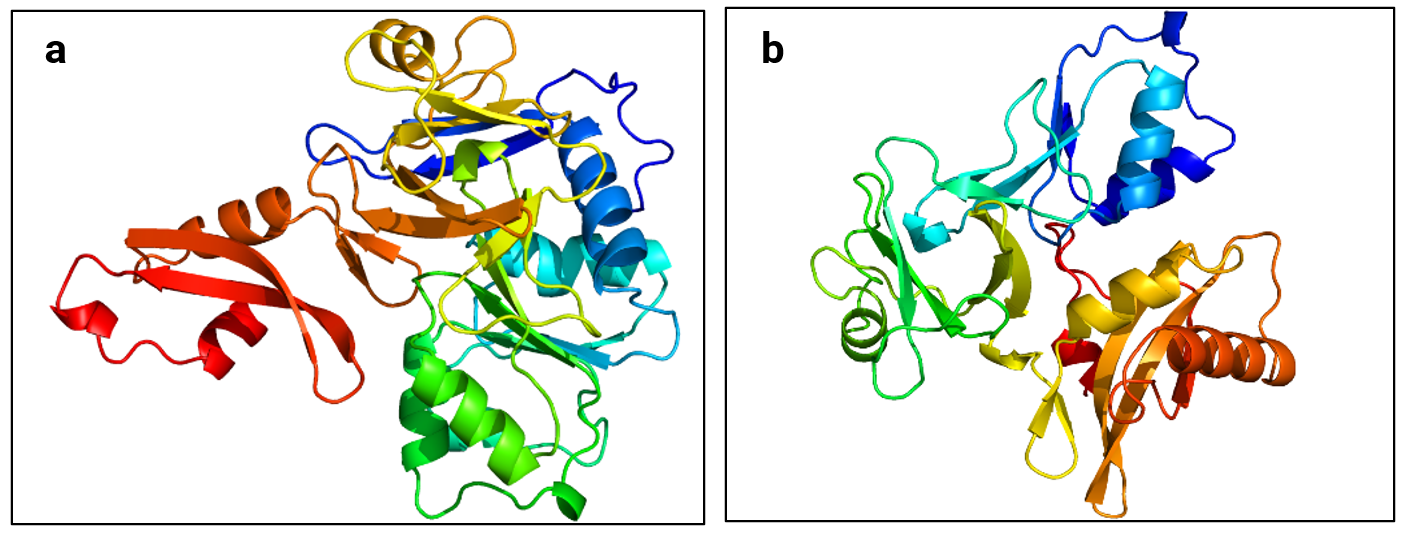


**Figure S48.** Visualization of the final predicted structure of enzyme (EC.6.2.1.25) in **a**. A. x. C2 and **b**. A. x. KW38.

**References**

[1] James J Davis, Alice R Wattam, Ramy K Aziz, Thomas Brettin, Ralph Butler, Rory M Butler, Philippe Chlenski, Neal Conrad, Allan Dickerman, Emily M Dietrich, Joseph L Gabbard, Svetlana Gerdes, Andrew Guard, Ronald W Kenyon, Dustin Machi, Chunhong Mao, Dan Murphy-Olson, Marcus Nguyen, Eric K Nordberg, Gary J Olsen, Robert D Olson, Jamie C Overbeek, Ross Overbeek, Bruce Parrello, Gordon D Pusch, Maulik Shukla, Chris Thomas, Margo VanOeffelen, Veronika Vonstein, Andrew S Warren, Fangfang Xia, Dawen Xie, Hyunseung Yoo, Rick Stevens, “PATRIC Bioinformatics Resource Center: expanding data and analysis capabilities | Nucleic Acids Research | Oxford Academic,” vol. Volume 48, Issue D1, p. Pages D606-D612, Jan. 2020, doi: doi.org/10.1093/nar/gkz943.

[2] Elliot Sollis, Abayomi Mosaku, Ala Abid, Annalisa Buniello, Maria Cerezo, Laurent Gil, Tudor Groza, Osman Güneş, Peggy Hall, James Hayhurst, Arwa Ibrahim, Yue Ji, Sajo John, Elizabeth Lewis, Jacqueline A L MacArthur, Aoife McMahon, David Osumi-Sutherland, Kalliope Panoutsopoulou, Zoë Pendlington, Santhi Ramachandran, Ray Stefancsik, Jonathan Stewart, Patricia Whetzel, Robert Wilson, Lucia Hindorff, Fiona Cunningham, Samuel A Lambert, Michael Inouye, Helen Parkinson, Laura W Harris, “NHGRI-EBI GWAS Catalog: knowledgebase and deposition resource | Nucleic Acids Research | Oxford Academic,” *Nucleic Acids Research*, vol. 51, no. D1, pp. D977–D985, Jan. 2023.

[3] L. Y. Geer *et al.*, “The NCBI BioSystems database,” *Nucleic Acids Res.*, vol. 38, no. suppl_1, pp. D492–D496, Jan. 2010, doi: 10.1093/nar/gkp858.

1. Million base pairs [↑](#footnote-ref-2)
2. Not available [↑](#footnote-ref-3)
3. Whole genome sequencing [↑](#footnote-ref-4)
4. Polychlorinated biphenyls [↑](#footnote-ref-5)
